# Enhancing Patient Activation and Self-Management Activities in Patients With Type 2 Diabetes Using the US Department of Defense Mobile Health Care Environment: Feasibility Study

**DOI:** 10.2196/17968

**Published:** 2020-05-26

**Authors:** Ronald W Gimbel, Lior M Rennert, Paul Crawford, Jeanette R Little, Khoa Truong, Joel E Williams, Sarah F Griffin, Lu Shi, Liwei Chen, LingLing Zhang, Jennie B Moss, Robert C Marshall, Karen W Edwards, Kristy J Crawford, Marie Hing, Amanda Schmeltz, Brandon Lumsden, Morgan Ashby, Elizabeth Haas, Kelly Palazzo

**Affiliations:** 1 Department of Public Health Sciences Clemson University Clemson, SC United States; 2 Nellis Family Medicine Residency Program Mike O'Callaghan Federal Hospital Las Vegas, NV United States; 3 Mobile Health Innovation Center Telemedicine & Advanced Technologies Research Center U.S. Army Medical Research & Materials Command Fort Gordon, GA United States; 4 Fielding School of Public Health University of California Los Angeles Los Angeles, CA United States; 5 College of Nursing and Health Sciences University of Massachusetts Boston Boston, MA United States; 6 Clinical Informatics Fellowship Program Madigan Army Medical Center Tacoma, WA United States; 7 Department of Internal Medicine Madigan Army Medical Center Tacoma, WA United States

**Keywords:** mHealth, diabetes mellitus, patient activation, patient-centered care, eHealth

## Abstract

**Background:**

Past mobile health (mHealth) efforts to empower type 2 diabetes (T2D) self-management include portals, text messaging, collection of biometric data, electronic coaching, email, and collection of lifestyle information.

**Objective:**

The primary objective was to enhance patient activation and self-management of T2D using the US Department of Defense’s Mobile Health Care Environment (MHCE) in a patient-centered medical home setting.

**Methods:**

A multisite study, including a user-centered design and a controlled trial, was conducted within the US Military Health System. Phase I assessed preferences regarding the enhancement of the enabling technology. Phase II was a single-blinded 12-month feasibility study that randomly assigned 240 patients to either the intervention (n=123, received mHealth technology and behavioral messages tailored to Patient Activation Measure [PAM] level at baseline) or the control group (n=117, received equipment but not messaging. The primary outcome measure was PAM scores. Secondary outcome measures included Summary of Diabetes Self-Care Activities (SDSCA) scores and cardiometabolic outcomes. We used generalized estimating equations to estimate changes in outcomes.

**Results:**

The final sample consisted of 229 patients. Participants were 61.6% (141/229) male, had a mean age of 62.9 years, mean glycated hemoglobin (HbA_1c_) of 7.5%, mean BMI of 32.7, and a mean duration of T2D diagnosis of 9.8 years. At month 12, the control group showed significantly greater improvements compared with the intervention group in PAM scores (control mean 7.49, intervention mean 1.77; *P*=.007), HbA_1c_ (control mean −0.53, intervention mean −0.11; *P*=.006), and low-density lipoprotein cholesterol (control mean −7.14, intervention mean 4.38; *P*=.01). Both groups showed significant improvement in SDSCA, BMI, waist size, and diastolic blood pressure; between-group differences were not statistically significant. Except for patients with the highest level of activation (PAM level 4), intervention group patients exhibited significant improvements in PAM scores. For patients with the lowest level of activation (PAM level 1), the intervention group showed significantly greater improvement compared with the control group in HbA_1c_ (control mean −0.09, intervention mean −0.52; *P*=.04), BMI (control mean 0.58, intervention mean −1.22; *P*=.01), and high-density lipoprotein cholesterol levels (control mean −4.86, intervention mean 3.56; *P*<.001). Significant improvements were seen in AM scores, SDSCA, and waist size for both groups and in diastolic and systolic blood pressure for the control group; the between-group differences were not statistically significant. The percentage of participants who were engaged with MHCE for ≥50% of days period was 60.7% (68/112; months 0-3), 57.4% (62/108; months 3-6), 49.5% (51/103; months 6-9), and 43% (42/98; months 9-12).

**Conclusions:**

Our study produced mixed results with improvement in PAM scores and outcomes in both the intervention and control groups. Structural design issues may have hampered the influence of tailored behavioral messaging within the intervention group.

**Trial Registration:**

ClinicalTrials.gov NCT02949037; https://clinicaltrials.gov/ct2/show/NCT02949037

**International Registered Report Identifier (IRRID):**

RR2-10.2196/resprot.6993

## Introduction

### Background

Type 2 diabetes (T2D) is a chronic disease with high rates of disability, impaired quality of life, and premature death [1-6]. The prevalence of T2D is increasing at an alarming rate in the United States; in 2017, the estimated number of patients was 30.3 million or about 9.4% of the adult population [2,3,7]. T2D is the leading cause of blindness, nontraumatic amputations, and adult renal failure [8], and it reduces life expectancy by 5 to 10 years [2]. On average, the medical expenditure of patients with T2D is about 2.3 times greater than that of a T2D-free individual [9]. The potential for mobile health (mHealth) technologies in the care of patients with T2D and other chronic conditions to date have assessed a variety of tools and techniques. Some of the most promising tools and techniques include regular collection of biometric devices (eg, glucometers, activity monitors [10,11], SMS messaging [10,12-16], secure email communication with clinical teams, and regular reporting of quality-of-life variables [17-20]). Each of these tools, used alone or in combination, has demonstrated varying degrees of effectiveness. In this study, we sought to incorporate several of the most promising mHealth capabilities in a patient-centered medical home (PCMH) workflow.

The concept of patient activation, detailed in our protocol [21], has been demonstrated to correlate with improved clinical outcomes, increased preventative care, and overall lower health care–related cost [22-25]. Research on increased activation and improved clinical outcomes using patient portal and personal health record (PHR)-based interventions have provided mixed results [16,26-33]. It is noteworthy that early manuscripts did not provide substantial detail related to the design and navigation of portal or PHRs or whether the embedded intervention included behavioral reinforcement. Several studies have demonstrated a relationship between increased patient activation and improved clinical outcomes (eg, hypertension, smoking, BMI, and glycated hemoglobin [HbA_1c_]) [5,16,25,34-38], whereas other studies did not achieve significant improvement in clinical outcomes [5,37,39]. There is evidence to suggest that activated patients are more likely to practice healthy behaviors related to their diet [37,40] and physical activity [37,41], better manage their T2D [37], and obtain preventive screenings [42].

### Objective

The primary goal of this research was to enhance patient activation and improve self-management of T2D using the US Department of Defense’s (DoD) Mobile Health Care Environment (MHCE) in a PCMH setting. We hypothesized that the MHCE intervention will lead to improvement in patient activation, increase in T2D self-care, and improvement in clinical outcomes.

## Methods

### Trial Design

The trial design and methods are described in detail elsewhere [21]. This was a feasibility study within the DoD’s Military Health System, which included a user-centered design phase and a feasibility trial conducted at two sites. In phase I, we assessed both patient and clinician preferences regarding MHCE technology capabilities and enhancements for T2D care. The phase II research was a single-blinded (patients only) 12-month feasibility study that incorporated randomization principles. We employed a 1:1 allocation ratio between the intervention and control groups.

The study was approved by the Institutional Review Boards (IRB) of Clemson University (#IRB2015-234) and the Madigan Army Medical Center (IRB #216073). The study was registered with ClinicalTrials.gov (NCT02949037) on October 31, 2016.

### Participant Enrollment

Patients were recruited from the PCMH clinic schedule, provider referrals, distributed posters and fliers, and population health databases. Potential participants were scheduled for a screening visit with a study staff member to establish eligibility, discuss and execute an informed consent document, and were administered the Patient Activation Measure (PAM) instrument. Patients’ PAM levels placed them in a stratified group, where patients were randomly allocated to intervention or control groups.

Clinicians practicing in the respective PCMH sites were invited to participate by the site’s principal investigator; this was a convenience sample. The clinician participants met with their senior research associate to review the IRB-approved minimal-risk information sheet. For phase II, clinicians signed an informed consent document. The clinician participants were not blinded to the study.

### Inclusion and Exclusion Criteria

Inclusion criteria for patient participation in phase I or II research included men and women (1) aged 18 years or older, (2) with the ability to understand and read English, (3) enrolled for primary care at one of the target PCMH sites, and (4) with a diagnosis of T2D. In addition, in phase II, we sought to recruit a maximum of 120 participants (per PCMH), with a proportional distribution of patients with PAM levels 1 through 4; a primer on PAM levels is included elsewhere [21,43]. As this is a feasibility study, we did not derive the 120 per site recruitment numbers from power calculations.

Inclusion criteria for clinician participation in phase I or II research included (1) physicians, physician assistants, nurse practitioners, or nurses at the target PCMHs and (2) providing care for patients with T2D.

Exclusion criteria for patient participation in phase I or II research included the following: (1) pregnant women; (2) non-English-speaking patients; (3) patients receiving hospice care; (4) patients having active cancer and receiving treatment with chemotherapy or radiation therapy; (5) patients taking warfarin; (6) patients that have been a recipient of gastric bypass or a similar procedure; (7) patients having a diagnosis of uncontrolled hypothyroidism; (8) patients having known Cushing syndrome; (9) patients being treated with oral steroids; (10) patients with known liver disease; (11) patients with a current diagnosis of cognitive impairments that would interfere with the use of technology; (12) patients having congestive heart failure, in New York Heart Association functional classification III or IV; and (13) patients unable to use a mobile device due to cognitive or physical impairments during initial screening.

Exclusion criteria for clinician participation in phase I or II research included the following: (1) not affiliated with the target site and (2) not providing care for patients with T2D.

### Setting and Site Selection

We sought to purposefully assess MHCE use in T2D care in two distinctly different PCMH environments and locations. The patient base included those on active duty, retirees, and dependents who have typically spent years in the military.

The Madigan Army Medical Center was the US Army’s second largest military treatment facility located in Tacoma, Washington, and was a tertiary facility with a level II trauma center and robust graduate medical education programs. They served a patient base of approximately 118,000 patients; about 7500 (or >6%) were living with T2D. Of the T2D population, about 15.00% (1125/7500) were active duty members or their dependents, and about 85.00% (6375/7500) were retirees and their dependents. Over half of the patients with T2D were aged 57-76 years. The study location within the medical center was an Internal Medicine PCMH with approximately 14,300 enrolled patients supported by a staff of 77 including 12 staff physicians and 8 residents.

The Mike O’Callaghan Federal Medical Center was a federal facility in the greater Las Vegas, Nevada area, which served approximately 47,000 patients; about 4500 (>9%) were living with T2D. Of the T2D population, about 4.00% (180/4500) were active duty members or their dependents, and about 96.00% (4320/4500) were retirees and their dependents. Over 72.00% (3240/4500) of the patients with T2D were aged ≥60 years. The study location within the medical center was a Family Medicine PCMH with approximately 7500 enrolled patients supported by a staff of 62 including 9 staff physicians and 26 residents.

### Description of the Mobile Health Care Environment

The DoD’s MHCE system is a secure health information system designed to support health services delivery and mHealth. The MHCE meets all physical and information security mandates, as prescribed by federal law and DoD regulation, for the protection of personal health information and personally identifiable information.

### Intervention Overview

The intervention has been described elsewhere [21]. This intervention enhanced MHCE in several ways. First, the intervention enhanced the capacity to include collection and visualization of data from Bluetooth-enabled medical devices. This included mapping data from device output into the MHCE, developing data visualization appropriate for mHealth and clinical care (eg, graphing outcomes and temporal trend patterns), migrating data to an analysis cell, and developing decision-support algorithms that drive safety alerts and behavioral message reinforcement. The devices used in this study included a Bluetooth-enabled scale, glucometer, blood pressure reader, and activity monitor. Second, the intervention expanded the capacity of the MHCE analysis cell to manage large amounts of data and to conduct both routine reports and research applications. Third, the intervention added patient activation and associated measurement instruments to capture baseline and ongoing changes to patient activation. Fourth, the intervention expanded the MHCE messaging platform that research associates used to send tailored behavioral messaging to patients in an effort to influence greater activation and reinforce positive behavior. An annotated visual presentation of patient screenshots and workflow is included in Multimedia Appendix 1.

The MHCE was accessed by mobile phones and tablets that used either an IOS or Android platform. The MHCE requires internet access for patients to sync data from devices to the MHCE backend portal, to receive tailored behavioral messages, or to support other functions accessible by clinicians (Multimedia Appendix 2). During the study, patients received SMS messages with hyperlinks to a separate secure information system platform used for the administration and analysis of PAM and Summary of Diabetes Self-Care Activities (SDSCA) instruments.

### Tailored Behavioral Messaging

A primary component of the MHCE system was tailored behavioral messaging. In this study, the research team developed behavioral messages tailored for each of the four PAM score levels; in total, we developed over 360 messages. The messages fell within 9 functional areas common to T2D care and the SDSCA survey: nutrition, home monitoring, physical activity, blood pressure, foot care, medications, smoking, glucose control, and general behavioral reinforcement. The message development process is outlined below (Textbox 1).

**Tailored behavioral messaging development process.**

A multistage message development process was guided by patient activation levels and theoretical constructs relevant to those levels. Specifically:

- Step 1: Health behavior researchers developed messages tailored for each of the four Patient Activation Measure (PAM) score levels and five type 2 diabetes self-care behaviors measured by the Summary of Diabetes Self-Care Activities.
- Step 2: Messages were assessed by two-person teams for content accuracy, reading level, and message appropriateness for the PAM level. Messages were then revised based on this review process. A clinical advisory team consisting of clinicians and researchers reviewed and approved the messages for clinical relevance and content.
- Step 3: A pretest (n=21) of the messages with a population similar to those targeted for the larger study was administered. Each person was provided with a random sample of five messages based on their PAM level. They were then asked four close-ended questions and one open-ended question about the messages.
- Step 4: Researchers reviewed PAM level–specific messages and rated them as acceptable, questionable, or unacceptable based on appropriateness for PAM level, behavioral theory construct, reading level, and content accuracy. Messages were then revised based on this review process.

This process supported triangulation review of findings that yielded a set of 360 PAM-level appropriate messages. At each step in the process, messages were reviewed and revised based on research team member feedback (stages 2 and 4) or by potential study participant feedback (stage 3). Overall, the first two steps in the process produced messages with a high degree of acceptability by people very similar to the study population. Pretest participants found the messages to be encouraging, useful, applicable, and impactful. This indicates a high level of credibility with regard to the application of the theoretical constructs within the messages.

For patients with baseline PAM level 1, we used messages addressing the emotional state of feeling overwhelmed and passive with an emphasis on the importance of taking action. PAM level 2 messages were designed to build knowledge and self-efficacy and focus on taking small steps that can be accomplished without much in-depth knowledge. PAM level 3 messages focused on building self-management skills, such as goal setting and self-monitoring. PAM level 4 messages focused on avoiding relapse when stressed.

In phase II of our study, tailored behavioral messages were delivered to each intervention group participant, via the MHCE accessed through their mobile device, based on both senior research associate–initiated and algorithm-automated schedules and thresholds developed according to PAM level, SDSCA responses, and agreed-upon general rotation. Senior research associates used the MHCE backend portal control panel for manual rotational scheduling of messages delivered 3 days per week. Participant responses to the SDSCA and data from synced devices triggered additional messaging if their clinical readings from biomedical devices exceeded the established thresholds. If a patient’s PAM level changed within the course of the study, the tailored behavioral messaging to that patient changed to align with the new PAM level.

### Phase I User-Centered Design

In phase I, we captured feedback from patients on MHCE navigation, use of external devices, ease of use, and satisfaction. We collected baseline research participant data following informed consent. One researcher led individual participants through usability testing, and an additional researcher-observer recorded observations using an observation guide for recording time on task, number of attempts by task, task sequences, and participant questions/comments. Following a researcher-provided demonstration of the MHCE, each participant was requested to concurrently navigate each component of the MHCE system via a mini tablet device under their control. For each task, each participant was asked three open-ended questions to evaluate task-specific user satisfaction. The researcher delivered a brief demonstration of the external devices used in the study. Participants were requested to (1) manually upload data, (2) sync each device with the app, and (3) interpret graphs. The data collection instrument used in phase I is shown in Multimedia Appendix 3.

Researchers have evaluated usability by applying definitions and evaluation metrics guided by the International Organization for Standardization’s 9241-11 usability framework and mHealth usability research [44]. Specific metrics to evaluate usability are effectiveness, efficiency, and satisfaction. Researchers used the Single Ease Question to evaluate informant satisfaction immediately after performing each task [45]. The system usability scale (SUS) was measured for overall informant satisfaction with the MHCE [46].

We assessed clinician preferences in phase I using focus groups at each study site. Two trained researchers facilitated focus groups using a semistructured interview guide to elicit feedback about the MHCE (Multimedia Appendix 4). A four-member team completed a thematically organized data analysis of the clinician and nurse feedback using an inductive narrative approach [47-49].

### Phase II Controlled Study: Patient Enrollment and Study Flow

In phase II, we aimed to recruit 240 patients (120 per site), with one half assigned to a control group. Eligible patients were assigned to four strata according to their PAM levels. After all patients were assigned, simple randomization was performed within each stratum to assign patients to either the MHCE (intervention) or control groups. Phase II research was conducted between July 2017 and November 2018.

### Mobile Health Care Environment Intervention Versus Intervention-Lite (Control)

Patients in both the intervention and intervention-lite (control) groups received the device package mentioned above. These devices collected and recorded biometric data; participants were trained on the biomedical device and peripheral equipment use.

For the intervention group, the devices were mapped to the MHCE system accessible from the patients’ mobile phone or an iPad mini tablet device. Data from their biomedical devices were visually presented in the MHCE with trend and scalable options. Safety algorithms were mapped to these clinical data to alert the participant and, depending on the measure, the clinical team when readings exceeded the established thresholds. The devices for the control group participants were not connected to the MHCE system.

### Initial Outcome Measures for Patient Component

Primary outcome measures included the PAM scores. Secondary outcome measures in the study were (1) SDSCA responses, (2) clinical measures (Textbox 1), (3) comorbid conditions, and (4) SUS survey scores.

### Patient Activation Measure Instrument

The self-reported PAM survey is associated with self-management behaviors, medication adherence, patient satisfaction, and quality of life [50,51]. Within a T2D-specific population, PAM is not related to knowledge regarding HbA_1c_ (the standard measure of average blood glucose level [52]), but it is associated with better glycemic control [53]. The PAM is a valid, reliable, unidimensional, and probabilistic Guttman-like scale instrument [43], and it is a standard tool to measure patient activation. We administered the PAM at screening visits in phases I and II and electronically every 3 months during phase II for both the intervention and control groups. In computing PAM in the analysis, we leveraged the 0-100 scoring mechanism based on licensing guidance from *Insignia Health*. PAM levels 1-4 are determined by the continuous PAM score.

### Summary of the Diabetes Self-Care Activities Instrument

The SDSCA instrument is a brief self-report instrument for measuring levels of self-management across different components of the T2D regimen [54]. The SDSCA includes 11 core items associated with T2D self-care. The SDSCA has been successfully used in numerous T2D studies both within and outside the United States [54-58]. The SDSCA has been validated and is considered a standard instrument in T2D care for measuring self-care activities [54]. We administered the SDSCA at the intake visit for phase II and electronically every 2 weeks during phase II for both the intervention and control groups.

### Clinical Measures

We collected clinical measures (Textbox 2) from patients at intake during phase I research. We collected and compared changes in clinical measures for both groups in phase II at 3 points: intake, midpoint (month six), and conclusion (month 12). Data were manually abstracted by senior research associates from the PHR. For patients assigned to the MHCE intervention group, the MHCE system recorded weight, systolic blood pressure, diastolic blood pressure, and blood glucose values to the MHCE module on a regular basis via Wi-Fi or Bluetooth-enabled peripheral equipment.

**Clinical measures in phase II.**

- Glycated hemoglobin
- Low-density lipoprotein
- High-density lipoprotein
- BMI
- Abdominal circumference
- Systolic blood pressure
- Diastolic blood pressure

### System Usability Scale Survey

The SUS survey is a 10-item Likert-like scaled survey used to convey a subjective assessment of system usability. The instrument was developed over 15 years ago and is used to measure the usability of websites [59]. In this study, we substituted the term “MHCE system” for the term “website” in the instrument. Phase I participants completed the SUS survey at the conclusion of the encounter. Phase II intervention group participants completed the SUS survey at the midpoint and study conclusion.

### Comorbid Conditions

We assessed and documented comorbid conditions among both the control and intervention groups during the prescreening of eligibility, at intake, at study midpoint, and at study conclusion. Although not primary outcome measures, any change in comorbid conditions was assessed.

### Data Analysis Strategy

We conducted the primary analyses for phase II using an intent-to-treat approach. Achievement of randomization was evaluated by comparing the baseline key variables between the MHCE intervention group and the control group. These baseline characteristics were compared using Kruskal-Wallis tests for continuous variables, Cochran-Armitage trend tests for ordinal variables, and chi-squared tests for binary variables and multinomial variables. Exact tests were performed to account for categorical variables with small cell counts.

Generalized estimating equations (GEE) with an autoregressive correlation structure were used to test the hypotheses that patients who participated in MHCE had higher PAM, SDSCA, and improved selected clinical outcomes and comorbid conditions than their control counterparts. The predictors in the model included a binary variable for a group, a three- or five-level categorical variable for time (depending on the number of repeated measurements for the outcome), and their interaction. All models included covariate adjustment for site, age, sex, race, duration of disease, work status, and baseline physical activity level. As the intervention group messages were tailored to each subject based on their baseline PAM level, we examined the effect of intervention within each baseline PAM level by including a three-way interaction between intervention, time, and baseline PAM level in the GEE models. CIs are reported to assess changes within groups. Within-group changes (from baseline) in outcomes are deemed significant at the alpha=.05 level if the estimated CIs exclude 0. Between-group comparisons of change (from baseline) in outcomes are deemed significant for two-sided *P* values< 0.5.

GEE were also used to test the hypothesis that patients who engaged at a higher rate with the interactive and tailored mobile technology in MHCE realized greater improvement in PAM level, SDSCA, and clinical measures. We defined the main independent variable for MHCE usage by an indicator variable set to 1 if the percentage of days engaged with MHCE in a 3-month period exceeded 80% (strong adherence). As adherence varies with time, the GEE model assumed working independence for the correlation structure [60]. All GEE analyses were conducted using the Generalized Linear Model procedure in the Statistical Analysis System.

## Results

### Phase I—User-Centered Design Research Participants

The research team recruited 10 research participants per site, with 20 participants for phase I. The study sample was 70% (14/20) male, 65% (13/20) white, middle-aged (average 62.8, range 40-82 years), obese (median BMI 33.7, range 24.3-43.5), with elevated blood sugar levels (median HbA_1c_ 7.2, range 5.8-12.2), and increased patient activation, 75% (15/20) with PAM level 3 scores at intake. Phase I research was conducted between November and December 2016.

### Phase I—Participant (Patients) Effectiveness With Mobile Health Care Environment Technology

Overall, participants performed better with goal-setting tasks (almost all completed these tasks without assistance), showed some difficulty with tasks that required manual entry (the majority could do so with little to no assistance), and had the most difficulty with Bluetooth syncing of devices (approximately half needed assistance or could not complete; Table 1).

**Table 1:** Phase I participant effectiveness with the technology. Here, n denotes the number of responses in each category.

Site and effectiveness		Blood pressure goal setting	Glucose goal setting	Weight goal setting	Steps goal setting	BP manual entry	BP sync with device	Glucose manual entry	Weight manual entry	Weight sync with device
**Madigan, n**										
	Completed without help	9	9	9	5	7	1	7	5	4
	Needed hints	1	0	1	4	3	7	3	5	3
	Failed to complete	0	1	0	1	0	2	0	0	3
**Nellis, n**										
	Completed without help	8	9	8	9	8	4	9	7	6
	Needed hint(s)	2	1	2	1	2	6	1	3	4
	Failed to complete	0	0	0	0	0	0	0	0	0
**Combined, n**										
	Completed without help	17	18	17	14	15	5	16	12	10
	Needed hint(s)	3	1	3	5	5	13	4	8	7
	Failed to complete	0	1	0	1	0	2	0	0	3

### Phase I—Participant (Patient) Efficiency With Mobile Health Care Environment Technology

Overall, goal setting and manual entry tasks took the least amount of time to complete, and Bluetooth syncing tasks took the most time (Table 2).

### Phase I—Participants and System Usability Survey Results

All phase I participants were administered a SUS related to MHCE use. The survey results were very favorable and related to the usability of MHCE. Specifically:

- SUS overall, Mean (SD): 83.8 (SD 14.9)=A+
- Usability sub factor, mean (SD): 86 (SD 13.4)=A+
- Learnability subfactor, mean (SD): 75 (SD 27.5)=A−

### Phase I—Results of the Qualitative Research Component (Patient Participants)

All participating participants indicated that the MHCE would “help them manage their diabetes” and give their health care provider a “better report of their health.” As expected, patients rated navigation tasks as “less difficult” and peripheral device tasks, for example, syncing, as “more difficult.” An overview of usability testing and patient feedback is included in Multimedia Appendix 5. Participants committed fewer errors with basic navigation tasks and more with peripheral devices. Patient participants suggested minor changes regarding the look and function of the app (Textbox 3).

### Phase I—Results of the Qualitative Research Component (Clinicians)

Overall, clinicians voiced a sense of optimism for the MHCE system, tempered with recommendations aimed at improving the patient experience, clinician adoption, and use of the data it generated. Clinician participants also suggested changes related to quality and safety (Textbox 4).

### Technology Adaptation Following Phase I Research and Before the Launch of Phase II

Patient and clinician suggestions were reviewed and incorporated as adaptations by our technology team within system constraints. A total of 29 specific recommendations were made to the technology team; 86% (25/29) of the recommendations were positively acted on and modifications occurred. Only 4 recommendations could not be supported (Textbox 5).

**Table 2:** Participant efficiency with technology.

Task	BP^a^ goal setting	Glucose goal setting	Weight goal setting	Steps goal setting	BP manual entry	BP sync with device	Glucose manual entry	Weight manual entry	Weight sync with device
Madigan, mean^b^ (SD)	55.02 (37.08)	19.83 (7.80)	40.18 (45.57)	61.31 (54.80)	48.00 (31.01)	121.74 (28.09)	32.09 (15.96)	54.09 (38.54)	65.08 (27.18)
Nellis, mean (SD)	51.53 (53.12)	30.29 (18.77)	19.02 (12.67)	19.91 (11.75)	46.44 (32.56)	103.04 (47.05)	30.10 (20.95)	35.37 (23.98)	68.09 (37.23)
Combined sites, mean (SD)	53.18 (45.00)	25.64 (15.50)	29.60 (34.31)	40.61 (44.04)	47.22 (30.97)	111.90 (39.37)	31.09 (18.15)	44.73 (32.69)	66.75 (32.25)

^a^BP: blood pressure.

^b^Mean denotes mean time in seconds.

**Participants’ suggestions for Mobile Health Care Environment improvement.**

- Suggestion #1: To increase the size of icons as they were difficult to see.
- Suggestion #2: To improve glucose graph responsiveness.
- Suggestion #3: To increase the font size for the Mobile Health Care Environment (MHCE) interface.
- Suggestion #4: To allow past dates for manual entry into the MHCE.

**Clinicians’ suggestions related to app safety alert functions.**

- Change default blood glucose entry to “Unclassified,” forcing patient entry of “after meal,” “bedtime,” or “fasting.”
- Define “after meal” glucose as >120 min post meal.
- Simplify safety alerts on the backend clinician portal.
- Add icons besides safety alerts.
- Add patient target ranges on graphs.

**Recommendations that could not be adopted.**

1. Desire for additional colors (limited colors were available).
2. Remove signal and refresh buttons (component of the base system).
3. Add alert icons (limited icon choices available).
4. Automatic syncing of devices (system could not support).

### Safety Algorithms Developed and Deployed in Phase II

The research team developed and implemented a series of safety alerts into the MHCE technology. These include thresholds and alerts for high/low blood glucose, high/low blood pressure, and weight loss/gain. Details on safety alerts are identified in a report attached as Multimedia Appendix 6.

### Phase II—Participant Randomization, Retention, and Comorbidities

A total of 240 participants met the study eligibility criteria and were randomized (117 to control group and 123 to intervention group). Of the 117 participants randomized to the control group, 5 withdrew before intake, 3 declined participation, and 2 did not meet the inclusion/exclusion criteria. Of the 123 participants randomized to the intervention group, 6 withdrew before intake, 3 declined participation, 1 did not meet the inclusion/exclusion criteria, and 2 did not provide reason. Thus, the final sample size consisted of 229 participants, with 112 randomized to the control group and 117 randomized to the intervention group.

Study retention and participation was high; 93.0% (213/229) of participants remained in the study through 12 months: 99.1% (111/112) in the control group and 86.3% (101/117) in the intervention group. One participant who withdrew from the control group cited a lack of ability to use the physical activity monitor as their primary reason for discontinuation. Of the 16 participants who did not complete the 12-month intervention, 8 cited technical issues with MHCE as their primary reason, 6 listed either a time constraint or relocation as their primary reason, and 2 were lost to follow-up. The specific technical issues cited by the 8 participants withdrawing from the study were not fully known. Anecdotal evidence received by the senior research associates suggested that a few patients were frustrated with system upgrades and challenges with occasional manual synching of devices.

Throughout the 12-month study, 20.6% (21/102) presented new comorbidities in the control group, and 11% (11/96) of subjects presented new comorbidities in the intervention group (χ^2^_1_=3.2; *P*=.07). On the basis of the covariate-adjusted GEE model, the estimated odds of a new comorbidity throughout the study was 2.27 times higher for the control group than for the intervention group (χ^2^_1_=3.9; *P*=.047).

### Phase II—Baseline Characteristics

Table 3 provides the study baseline demographics and outcomes at baseline. Participants had a mean age of 62.9 (SD 10.3) years, a mean BMI of 32.7 (SD 6.2), and were 61.6% (141/229) male. Mean HbA_1c_ was in the high range (mean 7.5%, SD 1.4%). The mean duration of T2D was 9.8 (SD 7.5) years. The study cohort was well educated; 87.8% (201/229) attended some college and 55.5% (127/229) had a college degree or higher. In this sample, 10% (23/229) of patients had a baseline PAM level of 1 (14/112, 12.5% in control group, 9/117, 7.7% in intervention group), 14.8% (34/229) had a baseline PAM level of 2 (19/112, 17.0% in control group, 15/117, 12.8% in intervention group), 51.5% (118/229) had a baseline PAM level of 3 (55/112, 49.1% in the control group, 63/117, 53.8% in the intervention group), and 23.6% (54/229) had a baseline PAM level of 4 (24/112, 21.4% in control group, 30/117, 25.6% in intervention group). Between group differences were not statistically significant (*P*=.13). The control and intervention groups were well balanced on most baseline characteristics, with the exception of work status (unemployment: 77/112, 68.8% in the control group, 59/117, 50.4% in the intervention group; full-time employment: 27/112, 24.1% in control group, 42/117, 35.9% in intervention group; *P*=.02), baseline low-density lipoprotein (LDL) cholesterol (control group: mean 97.1, SD 39.1; intervention group: mean 80.9 (SD 36.5); *P*=.001). Due to this imbalance, we adjust for these variables in all models.

**Table 3:** Characteristics of phase II participant demographics and outcomes at baseline.

Variable			Total	Group		*P* value
				Control	Intervention	
**Demographics**						
	Age (years), mean (SD)^a^		62.9 (10.3)	63.2 (9.8)	62.5 (10.8)	.49
	Gender (female), n (%)^b^		88 (38.4)	49 (43.8)	39 (33.3)	.11
	**Race, n (%)^c^**					.70
		American Indian	2 (0.9)	0 (0.0)	2 (1.7)	
		Asian	17 (7.4)	8 (7.1)	9 (7.7)	
		Black	33 (14.4)	14 (12.5)	19 (16.2)	
		Native Hawaiian	16 (7.0)	7 (6.3)	9 (7.7)	
		Unknown/unreported	18 (7.9)	8 (7.1)	10 (8.5)	
		White	143 (62.4)	75 (67.0)	68 (58.1)	
		Hispanic, n (%)^b^	24 (10.5)	10 (8.9)	14 (12.0)	.45
	**Education, n (%)^d^**					.22
		Less than high school	1 (0.4)	1 (0.9)	0.0 (0.0)	
		High school graduate	27 (11.8)	15 (13.4)	12 (10.3)	
		Some college, no degree	74 (32.3)	35 (31.3)	39 (33.3)	
		Associate’s degree	41 (17.9)	24 (21.4)	17 (14.5)	
		Bachelor’s degree	46 (20.1)	20 (17.9)	26 (22.2)	
		Master’s degree	36 (15.7)	16 (14.3)	20 (17.1)	
		Professional degree	4 (1.7)	1 (0.9)	3 (2.6)	
	Weight (lbs), mean (SD)^a^		210 (47.1)	209 (50.1)	211 (44.2)	.50
	Height (inches), mean (SD)^a^		67.1 (4.0)	67.0 (3.8)	67.2 (4.2)	.44
	Duration of type 2 diabetes (years), mean (SD)^a^		9.8 (7.5)	10.0 (7.6)	9.6 (7.4)	.69
	Current smoker, n (%)^b^		15 (6.6)	8 (7.1)	7 (6.0)	.72
	Hospitalization, n (%)^b^		34 (14.8)	21 (18.8)	13 (11.1)	.10
	Emergency room visit, n (%)^b^		87 (38.0)	45 (40.2)	42 (35.9)	.50
	Surgeries, n (%)^b^		31 (13.5)	14 (12.5)	17 (14.5)	.65
	**Work status, n (%)^c^**					.02
		Unemployed	136 (59.4)	77 (68.8)	59 (50.4)	
		Part-time	24 (10.5)	8 (7.1)	16 (13.7)	
		Full-time	69 (30.1)	27 (24.1)	42 (35.9)	
	Family history of cancer, n (%)^b^		137 (59.8)	72 (64.3)	65 (55.6)	.18
	Family history of heart disease, n (%)^b^		132 (57.6)	64 (57.1)	68 (58.1)	.88
	Family history of high Blood pressure, n (%)^b^		164 (71.6)	78 (69.6)	86 (73.5)	.52
	Family history of high cholesterol, n (%)^b^		111 (48.5)	55 (49.1)	56 (47.9)	.85
	Site=Madigan, n (%)^b^		113 (49.3)	51 (45.5)	62 (53.0)	.26
	**PAM^e^, n (%)^d^**					.13
		Level 1	23 (10.0)	14 (12.5)	9 (7.7)	
		Level 2	34 (14.8)	19 (17.0)	15 (12.8)	
		Level 3	118 (51.5)	55 (49.1)	63 (53.8)	
		Level 4	54 (23.6)	24 (21.4)	30 (25.6)	
**Outcomes**						
	PAM, mean (SD)^a^		63.2 (12.8)	62.8 (13.5)	63.6 (12.2)	.56
	Summary of Diabetes Self-Care Activities score, mean (SD)^a^		37.5 (14.0)	38.0 (14.3)	37.1 (13.8)	.58
	Glycated hemoglobin, mean (SD)^a^		7.5 (1.4)	7.6 (1.6)	7.5 (1.3)	.95
	BMI, mean (SD)^a^		32.7 (6.2)	32.6 (6.8)	32.8 (5.7)	.77
	Waist (inches), mean (SD)^a^		44.1 (5.9)	43.9 (6.4)	44.2 (5.4)	.63
	Diastolic BP (mm Hg), mean (SD)^a^		79.5 (9.6)	80.7 (9.4)	78.4 (9.7)	.09
	Systolic BP (mm Hg), mean (SD)^a^		133 (17.6)	134 (17.9)	131 (17.3)	.25
	High-density lipoprotein cholesterol (mg/dL), mean (SD)^a^		45.2 (13.6)	46.6 (15.4)	43.8 (11.3)	.47
	Low-density lipoprotein cholesterol (mg/dL), mean (SD)^a^		88.9 (38.6)	97.1 (39.1)	80.9 (36.5)	.001

^a^Continuous variables compared using Wilcoxon/Kruskal-Wallis test.

^b^Binary variables compared using chi-square test.

^c^Nominal variables compared using Fisher exact test.

^d^Ordinal variables compared using Cochran-Armitage trend test.

^e^PAM: Patient Activation Measure.

### Phase II—Primary Outcomes

Within- and between-group comparisons based on GEE are displayed in Table 4. For each intervention group, we assessed changes in the outcome between baseline and month 6 and baseline and month 12. For each group, CIs that exclude 0 indicate a significant change in the outcome between baseline and a given month. *P* values <.05 indicate that this change in the outcome was significantly different between the intervention and control groups. At months 6 and 12, statistically significant improvements were seen in the control group for PAM (month 6: control group improvement=4.81, 95% CI 2.21 to 7.42; month 12: control group improvement=7.49, 95% CI 4.44 to 10.55). That is, improvement in PAM for the control group was 4.81 between baseline and month 6 and 7.49 between baseline and month 12. Although the intervention group showed slight improvement in PAM, these improvements were not statistically significant (month 6: intervention group improvement=1.57, 95% CI −1.34 to 4.48; month 12: intervention group improvement=1.77, 95% CI −1.02 to 4.57). The improvement in the control group was significantly greater than the improvement in the intervention group at month 12 only (month 6: between-group difference=3.24; *P*=.10 and month 12: between-group difference=5.72; *P*=.007).

For each group, estimates (and the corresponding CIs) are given for the *change* in outcome between month 6 and baseline and month 12 and baseline. CIs that exclude 0 indicate statistically significant within-group change between a given month (ie, month 6 or 12) and baseline. The first *P* value column corresponds to testing whether the change in outcomes between month 6 and baseline is different between the 2 groups. The second *P* value column corresponds to testing whether the change in outcomes between month 12 and baseline is different between the 2 groups.

**Table 4:** Analysis of change from baseline at midpoint and study end.

Outcome	Month 6			Month 12		
	Control, OR^a^ (95% CI)	Intervention, OR (95% CI)	*P* value	Control, OR (95% CI)	Intervention, OR (95% CI)	*P* value
Patient Activation Measure score	4.81 (2.21 to 7.42)	1.57 (−1.34 to 4.48)	.10	7.49 (4.44 to 10.55)	1.77 (−1.02 to 4.57)	.007
Summary of Diabetes Self-Care Activities score	7.52 (5.43 to 9.61)	7.52 (5.30 to 9.73)	>.99	6.97 (4.40 to 9.55)	7.52 (5.06 to 9.97)	.77
Glycated hemoglobin	−0.36 (−0.57 to −0.14)	−0.18 (−0.36 to 0.01)	.21	−0.53 (−0.78 to −0.29)	−0.11 (−0.28 to 0.07)	.006
BMI (kg/m^2^)	−0.31 (−0.62 to 0.00)	−0.28 (−0.53 to −0.02)	.86	−0.45 (−0.82 to −0.07)	−0.36 (−0.62 to −0.10)	.72
Waist (inches)	−0.57 (−0.96 to −0.19)	−0.76 (−1.09 to −0.44)	.46	−1.46 (−1.95 to −0.98)	−1.49 (−1.90 to −1.07)	.95
Diastolic blood pressure (mm Hg)	−1.83 (−3.81 to 0.15)	−0.58 (−2.30 to 1.14)	.35	−2.18 (−3.98 to −0.38)	−1.72 (−3.22 to −0.22)	.70
Systolic blood pressure (mm Hg)	−2.90 (−6.31 to 0.52)	0.57 (−3.13 to 4.26)	.18	−2.88 (−6.63 to 0.87)	−1.63 (−4.99 to 1.74)	.63
High-density lipoproteincholesterol (mg/dL)	1.22 (−0.12 to 2.57)	1.59 (−0.39 to 3.58)	.76	−1.39 (−2.82 to 0.03)	−0.26 (−2.26 to 1.74)	.37
Low-density lipoprotein cholesterol (mg/dL)	−5.78 (−11.22 to −0.33)	0.12 (−5.69 to 5.93)	.15	−7.14 (−13.43 to −0.85)	4.38 (−2.16 to 10.91)	.01

^a^OR: odds ratio.

### Phase II—Secondary Outcomes

At month 6, the control group exhibited statistically significant improvements in HbA_1c_ and LDL cholesterol (as indicated by the CIs in the first column of Table 4). Both intervention and control groups exhibited statistically significant improvements in SDSCA, BMI, and waist size. However, the differences between the intervention and control groups on improvements in these outcomes were not significantly different at month 6 (as indicated by *P* values >.05 in the third column of Table 4). At month 12, statistically significant improvements occurred in SDSCA, BMI, waist size, and diastolic blood pressure in both the intervention and control groups. The control group also exhibited significant improvements in HbA_1c_ and LDL cholesterol at month 12, and these improvements were significantly greater compared with those of the intervention group (HbA_1c_: between-group difference=−0.42; *P*=.006 and LDL cholesterol: between-group difference=−11.52; *P*=.01).

### Phase II—Stratified Analyses by Baseline Patient Activation Measure Level

The intervention is tailored according to baseline PAM level, and therefore, we expect the effect of intervention over time to differ by subjects’ baseline PAM level. To test this, a three-way interaction term for the intervention group, time, and baseline PAM level was incorporated into the above GEE models. The results for the change between baseline and end of study (month 12) for each group, stratified by baseline PAM level, are presented in Tables 5 and 6.

Within each baseline PAM level, estimates (and corresponding CIs) are given for the *change* in outcome between month 12 and baseline for both the intervention and control groups. CIs that exclude 0 indicate statistically significant within-group change between month 12 and baseline for subjects in a given PAM level. The *P* value column corresponds to testing whether the change in outcomes is different between the intervention and control groups within a given PAM level.

Among subjects with baseline PAM level 1, both intervention and control groups exhibited significant improvements in PAM score at month 12 (control group improvement=20.38, 95% CI 13.05 to 27.71; intervention group improvement=15.93, 95% CI 8.99 to 22.87); however, between-group differences did not reach statistical significance (*P*=.39). On secondary outcomes, the control group exhibited significant improvements in waist size, diastolic, and systolic blood pressure. The intervention group exhibited significant improvements in SDSCA, HbA_1c_, BMI, waist size, and HDL cholesterol. Statistically significant between-group differences occurred only in HbA_1c_, BMI, and HDL cholesterol, indicating that the intervention group exhibited greater improvement in these outcomes compared with the control group (HbA_1c_: between-group difference=−0.43, *P*=.04; BMI: between-group difference=−1.80, *P*=.01; HDL cholesterol: between-group difference=8.42, *P*<.001).

**Table 5:** Analysis of change from baseline to study end by baseline Patient Activation Measure level.

Outcome	Baseline PAM^a^ level 1			Baseline PAM level 2		
	Control, OR^b^ (95% CI)	Intervention, OR (95% CI)	*P* value	Control, OR (95% CI)	Intervention, OR (95% CI)	*P* value
PAM score	20.38 (13.05 to 27.71)	15.93 (8.99 to 22.87)	.39	13.09 (6.26 to 19.92)	7.66 (3.33 to 11.99)	.19
Summary of Diabetes Self-Care Activities score	8.99 (−1.49 to 19.48)	12.78 (3.71 to 21.85)	.59	6.42 (0.49 to 12.36)	11.09 (6.84 to 15.34)	.21
Glycated hemoglobin	−0.09 (−0.33 to 0.16)	−0.52 (−0.87 to −0.18)	.04	−1.04 (−1.88 to −0.20)	−0.09 (−0.59 to 0.42)	.06
BMI (kg/m^2^)	0.58 (−0.37 to 1.53)	−1.22 (−2.21 to −0.23)	.01	−0.54 (−1.28 to 0.20)	0.13 (−0.63 to 0.90)	.22
Waist (inches)	−1.50 (−2.73 to −0.27)	−1.98 (−3.32 to −0.64)	.61	−1.42 (−2.36 to −0.47)	−1.90 (−3.30 to −0.51)	.57
Diastolic blood pressure (mm Hg)	−4.00 (−7.86 to −0.14)	−3.89 (−10.79 to 3.01)	.98	−3.22 (−7.38 to 0.95)	−0.50 (−4.18 to 3.18)	.34
Systolic blood pressure (mm Hg)	−9.93 (−17.18 to −2.68)	−8.33 (−17.17 to 0.51)	.78	5.86 (−3.95 to 15.67)	−2.42 (−10.76 to 5.92)	.21
High-density lipoprotein cholesterol (mg/dL)	−4.86 (−8.16 to −1.56)	3.56 (0.50 to 6.61)	<.001	1.40 (−3.22 to 6.02)	−2.76 (−6.30 to 0.77)	.16
Low-density lipoprotein cholesterol (mg/dL)	−10.21 (−25.74 to 5.31)	9.56 (−10.02 to 29.13)	.12	−5.62 (−24.94 to 13.70)	10.55 (−12.21 to 33.30)	.29

^a^PAM: Patient Activation Measure.

^b^OR: odds ratio.

**Table 6:** Analysis of change from baseline to study end baseline Patient Activation Measure level (continued).

Outcome	Baseline PAM^a^ level 3			Baseline PAM level 4		
	Control, OR^b^ (95% CI)	Intervention, OR (95% CI)	*P* value	Control, OR (95% CI)	Intervention, OR (95% CI)	*P* value
PAM score	6.29 (2.24 to 10.35)	4.26 (0.88 to 7.64)	.45	−1.44 (−7.39 to 4.50)	−10.49 (−16.54 to −4.44)	.04
Summary of Diabetes Self-Care Activities score	6.49 (2.75 to 10.23)	7.58 (4.44 to 10.73)	.66	7.02 (3.15 to 10.89)	3.95 (−1.76 to 9.66)	.38
Glycated hemoglobin	−0.49 (−0.83 to −0.16)	−0.04 (−0.25 to 0.16)	.02	−0.57 (−1.11 to −0.03)	−0.09 (−0.55 to 0.36)	.18
BMI (kg/m^2^)	−0.43 (−0.86 to 0.01)	−0.42 (−0.76 to −0.07)	.97	−1.25 (−2.47 to −0.04)	−0.09 (−0.53 to 0.35)	.08
Waist (inches)	−1.51 (−2.18 to −0.83)	−1.56 (−2.08 to −1.03)	.91	−1.37 (−2.63 to −0.10)	−0.90 (−1.71 to −0.08)	.54
Diastolic blood pressure (mm Hg)	−2.55 (−5.22 to 0.11)	−1.23 (−3.24 to 0.79)	.44	−0.19 (−4.24 to 3.86)	−2.22 (−5.41 to 0.96)	.44
Systolic blood pressure (mm Hg)	−4.33 (−9.20 to 0.53)	−1.58 (−6.34 to 3.19)	.43	−2.78 (−12.44 to 6.89)	1.69 (−5.17 to 8.56)	.46
High-density lipoprotein cholesterol (mg/dL)	−0.93 (−2.84 to 0.99)	0.12 (−2.56 to 2.81)	.53	−2.44 (−4.91 to 0.02)	−1.03 (−6.10 to 4.05)	.62
Low-density lipoprotein cholesterol (mg/dL)	−3.99 (−12.36 to 4.37)	3.32 (−3.62 to 10.26)	.19	−13.38 (−27.02 to 0.25)	−0.19 (−17.09 to 16.70)	.23

^a^PAM: Patient Activation Measure.

^b^OR: odds ratio.

Among subjects with baseline PAM levels 2 and 3, both the intervention and control groups exhibited significant improvements in PAM, SDSCA, and waist size. However, between-group differences were not statistically significant. The control group also exhibited significant improvements in HbA_1c_; between-group differences were significant among those with baseline PAM level 3 only (between-group difference=−0.45; *P*=.02). The intervention group also exhibited significant improvement in BMI among subjects with PAM level 3, but the between-group differences did not reach statistical significance.

Among subjects with baseline PAM level 4, the intervention group exhibited a significant decrease in PAM (intervention group change=−10.49; 95% CI −16.54 to −4.44), whereas control subjects exhibited no significant change (control group change=−1.44; 95% CI −7.39 to 4.50). This difference in change was statistically significant (between-group difference=−9.05; *P*=.04). The control group exhibited significant improvements in SDSCA, HbA_1c_, and BMI. Both groups exhibited significant improvements in waist size. However, between-group differences did not reach statistical significance in these outcomes.

### Phase II—Participant Engagement and Usability

The percentage of subjects who engaged with MHCE at least 50% of the days in each time period was 60.7% (68/112) for months 0-3, 57.4% (62/108) for months 3-6, 49.5% (51/103) for months 6-9, and 43% (42/98) for months 9-12. The percentage of subjects who engaged with MHCE at least 80% of the days in each time period was 41.1% (46/112) for months 0-3, 41.7% (45/108) for months 3-6, 14.6% (15/103) for months 6-9, and 26% (25/98) for months 9-12. Strong adherence, defined engagement with MHCE on at least 80% of days in each time period, was associated with improvement in all outcomes. However, only the association with HbA_1c_ reached statistical significance (mean improvement=0.35; *P*=.049).

The mean SUS score for the MHCE group was 75.6 (SD 17.3) at month 6 and 76.8 (SD 14.8) at month 12. Strong adherence was associated with a higher SUS score (average increase=5.01; *P*=.04). As SUS was not recorded for the control group, group differences could not be compared.

## Discussion

### Principal Findings

The primary goal of this large feasibility study was to enhance the PAM level and improve self-management of T2D care using the DoD’s interactive and tailored MHCE system for T2D in a PCMH setting. To this end, we hypothesized that a user-centered design process would successfully shape the development of the MHCE for use in the self-management of T2D care. The research team engaged both patients and clinicians, observing patient and clinician overall satisfaction with the MHCE, high SUS (A+ and A−) scores, and 29 offered recommendations for system improvement; 86% (25/29) of these recommendations resulted in intervention modifications before feasibility testing. The methods and findings from the study’s phase I research align well with the aging barriers addressed by Wildenbos et al [61]. These include cognitive, physical ability, perception, and motivational barriers.

When comparing this study with comparable mHealth studies, there were differences in participant demographics. Specifically, our research participants tended to be older (age: mean 62.9 years, SD 10.3) than participants in other studies [36,38,62-69] and had a larger BMI (32.7 kg/m^2^, SD 6.2 kg/m^2^) at baseline [62,64-66,68]. This study engaged a substantial percentage, 59.4% (136/229), of unemployed participants; most of them were military retirees. Finally, our research participants were members of a PCMH site within one of two military treatment facilities (ie, military run hospitals) and as such typically received health care services with no (or minimal) out-of-pocket costs. A descriptive primer on this federal health system has been published [70].

Overall retention was high in this study, 93.0% (213/229) for the 12-month study, which is consistent with other mHealth projects [66] and demonstrates the feasibility of conducting a PCMH intervention among T2D patients. The 12-month duration of this study was longer than loosely comparable studies, which generally targeted a 9-month [36,63], 6-month [64,65], or 3-month duration [38,62].

The team hypothesized that the use of the MHCE would increase the PAM and self-care activities of patients with T2D and that patients who engage at a higher rate with the MHCE would realize improvement in clinical measures. In our study, both intervention and intervention-lite (control) groups showed significant improvement from baseline to 12 months in SDSCA (measuring self-care activities) as well as in BMI, waist size, and diastolic blood pressure control. A somewhat surprising finding in the study is that only the control group exhibited significant improvement in the PAM score. Furthermore, improvements in the control group exceeded improvements in the intervention group for most clinical outcomes.

However, a closer analysis of the data reveals a complex yet intriguing pattern of how mHealth intervention effectiveness can vary by the baseline level of patient activation. With the exception of patients with the highest level of activation (PAM level 4), all intervention group patients exhibited significant improvements in the PAM score. Furthermore, the intervention appears to be superior to control for patients with the lowest level of activation (PAM level 1). In this class of patients, the intervention group showed significantly greater improvements at the end of the study in HbA_1c_, BMI, and HDL cholesterol levels compared with the control group (these were the only significant differences between the groups). This finding may suggest that in a resource-limited health system environment, a targeted investment in mHealth to support self-management of T2D for PAM level 1 patients may yield optimal results compared with patients who are more activated. With the more activated patient, demonstrating higher PAM scores, a less costly peripheral device (eg, glucometer, activity monitor, and scale) may by itself enhance self-care activities.

Our finding that the intervention group exhibited significant improvements over the control group in several cardiovascular outcomes in T2D patients with baseline PAM level 1 is consistent with previous findings that text messaging is effective in helping patients with T2D achieve better glycemic control [16,36,71]. Although tailored messaging did not generate superior results compared with the control group for patients with higher PAM levels, it does not suggest that the PAM-aligned tailored behavioral messaging was faulty or should be discarded. In our study, the DoD technology partners secured the tailored behavioral messaging embedded within the MHCE under an indicator labeled as “health tips.” However, because of privacy concerns, patients could only see the message after logging into the MHCE mobile app through its security layers to view the message. The multistep process of accessing the messages was indirect and could have been overlooked on the patients’ personal cell phone. Future efforts should consider redesigning the MHCE (or mHealth platforms) to accommodate nonpersonal health information (PHI) and tailored health message delivery to the patient in his/her preferred communication process. Most of this will likely be via mobile app push notifications and/or text messaging. Several published studies have successfully leveraged tailored health messaging (without PHI), delivered in a direct delivery process via text messaging to participants [16,36,69]; one study was very short term in nature [69].

Although SUSs remained constant throughout the study and were associated with increased patient engagement, overall patient engagement with the MHCE system decreased throughout the study period. Decreased engagement may be partly explained by frustration with system upgrades and synching of devices expressed by some patients, along with the multistep process of MHCE discussed above. However, high levels of initial patient engagement in other mHealth projects that drop off after 6 to 12 months have been observed [16,63,72]. This might suggest that systems such as the MHCE may be optimally deployed in self-care of T2D for periods up to about 6 months; lengthy engagement (>6 months) may not generate the desired results.

### Limitations

Our study design is not immune from potential threats to validity. Both the intervention and control groups were issued the same clinical outcome measuring devices and completed the biweekly SDSCA survey. However, we do not have information on whether patients in the control group did the synchronization of their measuring devices to their mobile phones, tablet computers, or desktop/laptop computers for viewing and tracking purposes. Therefore, we cannot rule out the possibility that a substantial proportion of the control group had access to visualized tracking information similar to the intervention group. For these patients, improvement in these outcomes may be explained by the same mechanisms whereby self-weighing is linked with weight loss [73]. Another limitation of the study is that food intake and daily activities outside the research sites are uncontrolled due to the nature of this type of field experiment.

Finally, although our study included user-center design testing (including solicitation of user design preferences) and ultimately generated a high retention rate for the 12-month study, patient engagement with MHCE decreased throughout the course of the phase II study. This trend was also seen in previous mHealth projects, and future studies should therefore consider incorporating follow-up participant assessments of continued engagement. If implemented following phase I, this may help the understanding of discontinued engagement and provide opportunities for improvement.

## Appendix

Multimedia Appendix 1 Annotated visual presentation of patient screenshots and workflow.

Multimedia Appendix 2 Annotated visual presentation of clinician access.

Multimedia Appendix 3 Phase I data collection instrument.

Multimedia Appendix 4 Clinician focus group - facilitator's guide.

Multimedia Appendix 5 Mobile Health Care Environment modifications report to funder.

Multimedia Appendix 6 Standards, alerts and safety algorithms report.

## Abbreviations

DoD: US Department of Defense
GEE: generalized estimating equations
HbA_1c_: glycated hemoglobin
LDL: low-density lipoprotein
MHCE: Mobile Health Care Environment
mHealth: mobile health
PAM: Patient Activation Measure
PCMH: patient-centered medical home
PHI: personal health information
PHR: personal health record
SDSCA: Summary of Diabetes Self-Care Activities
SUS: System Usability Scale
T2D: type 2 diabetes
