# Supplementary material for: Enhancing Patient Activation and Self-Management Activities in Patients With Type 2 Diabetes Using the US Department of Defense Mobile Health Care Environment: Feasibility Study
Source: J Med Internet Res. 2020 May 26;22(5):e17968. doi: 10.2196/17968 (PMC7284404; doi:10.2196/17968)
Supplement: Multimedia Appendix 1 [file jmir_v22i5e17968_app1.pptx]

## Slide 1
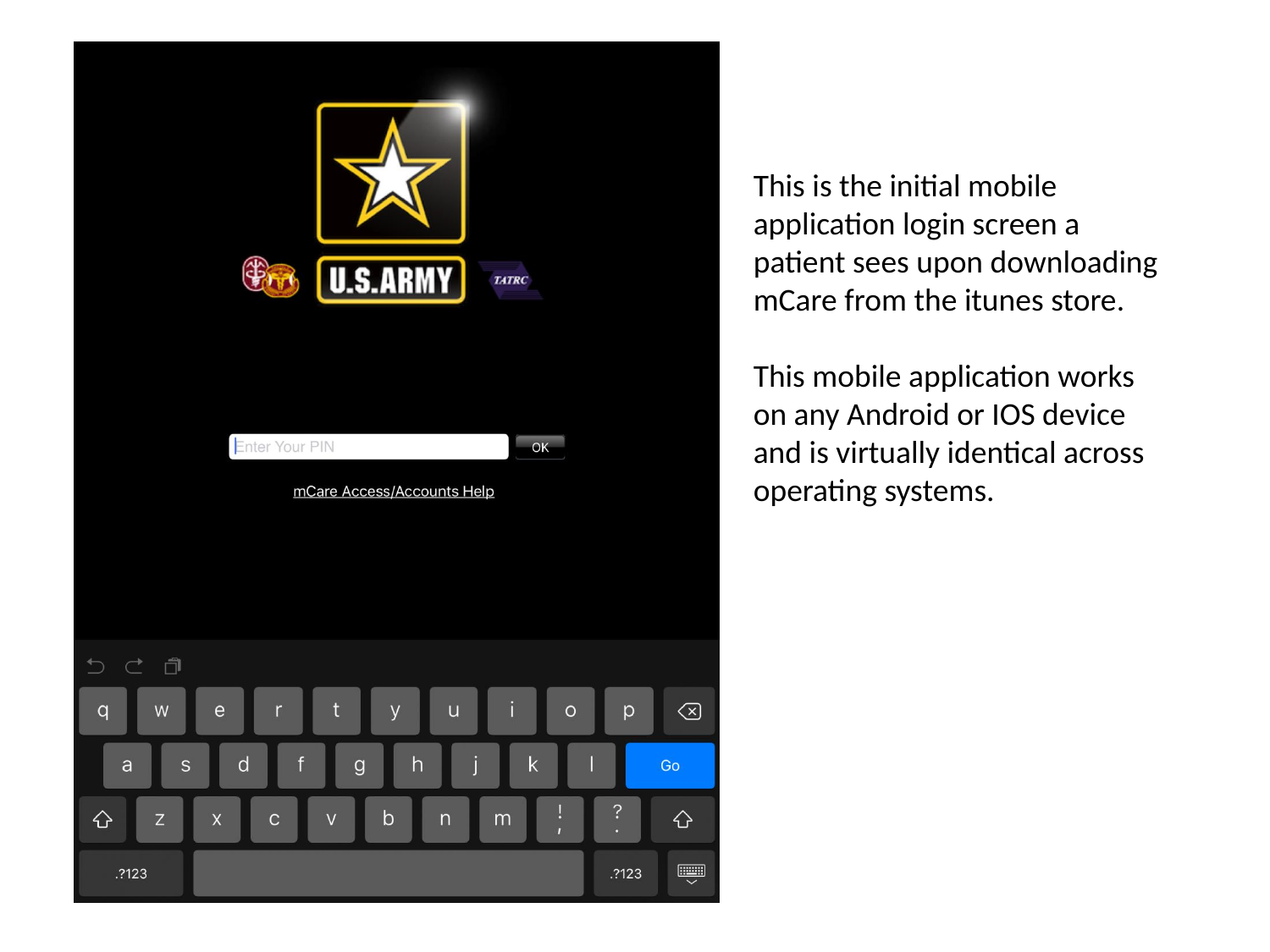

This is the initial mobile application login screen a patient sees upon downloading mCare from the itunes store.
This mobile application works on any Android or IOS device and is virtually identical across operating systems.

## Slide 2
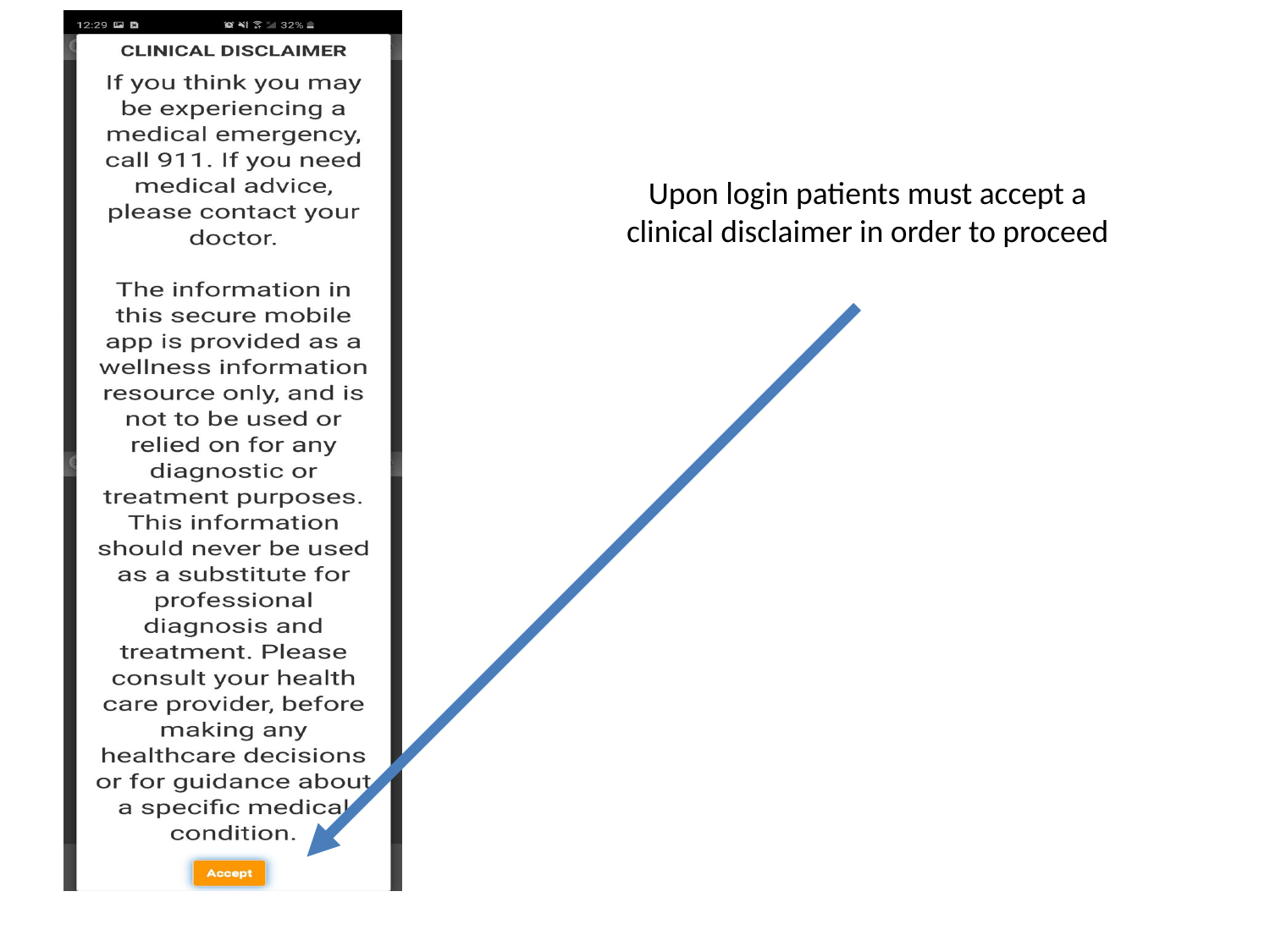

Upon login patients must accept a clinical disclaimer in order to proceed

## Slide 3
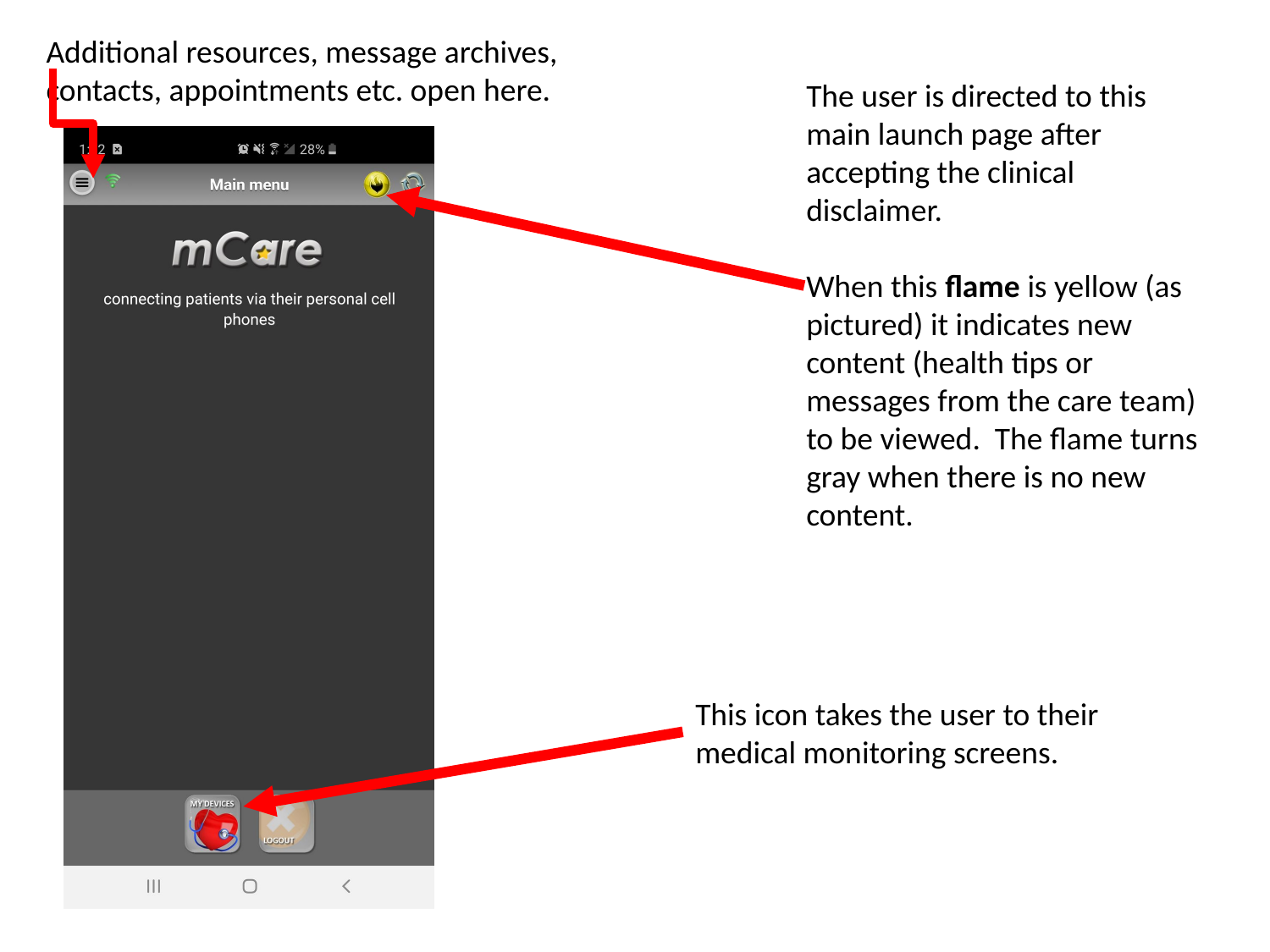

Additional resources, message archives, contacts, appointments etc. open here.
The user is directed to this main launch page after accepting the clinical disclaimer.
When this flame is yellow (as pictured) it indicates new content (health tips or messages from the care team) to be viewed. The flame turns gray when there is no new content.
This icon takes the user to their medical monitoring screens.

## Slide 4
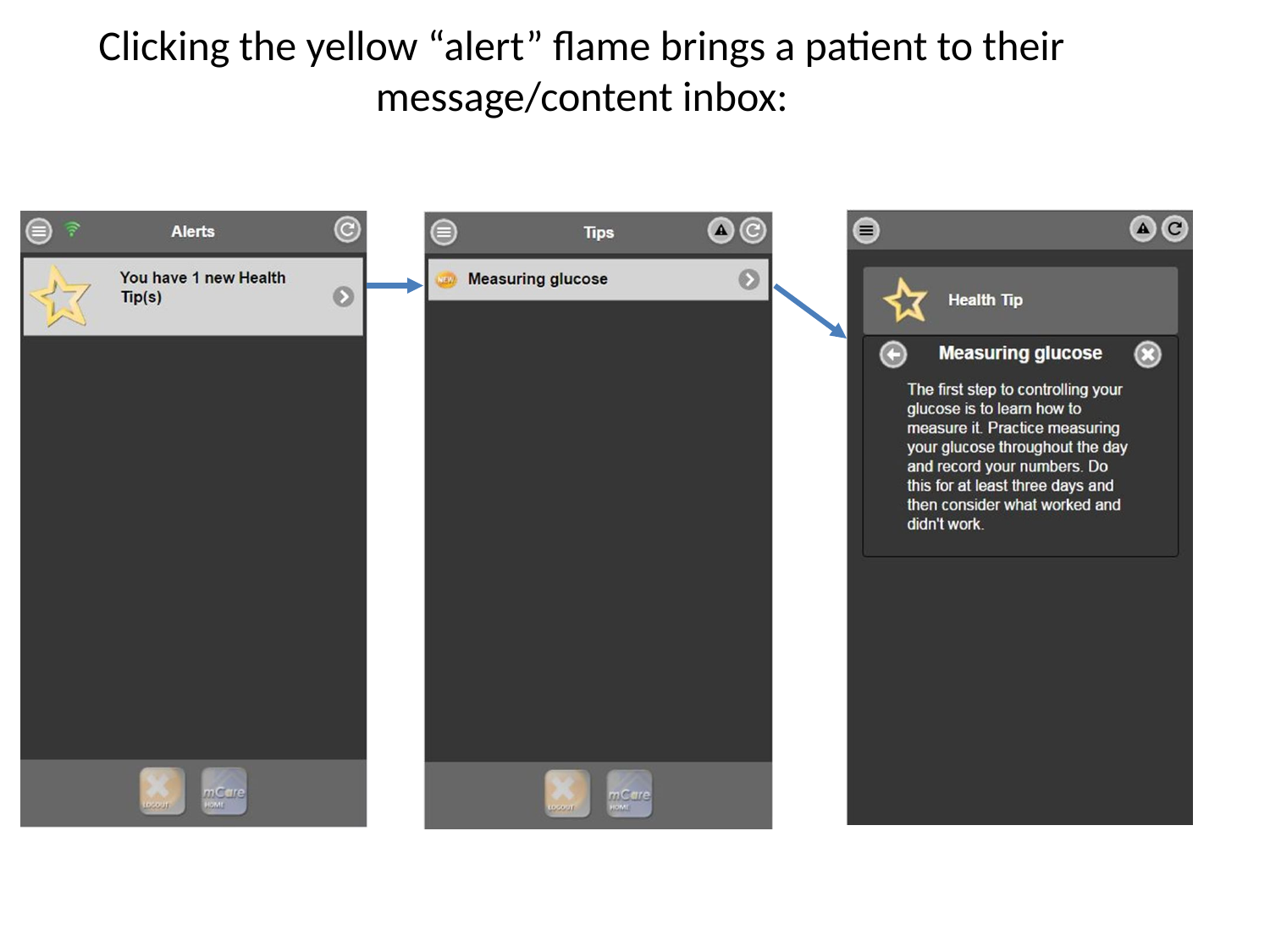

# Clicking the yellow “alert” flame brings a patient to their message/content inbox:

## Slide 5
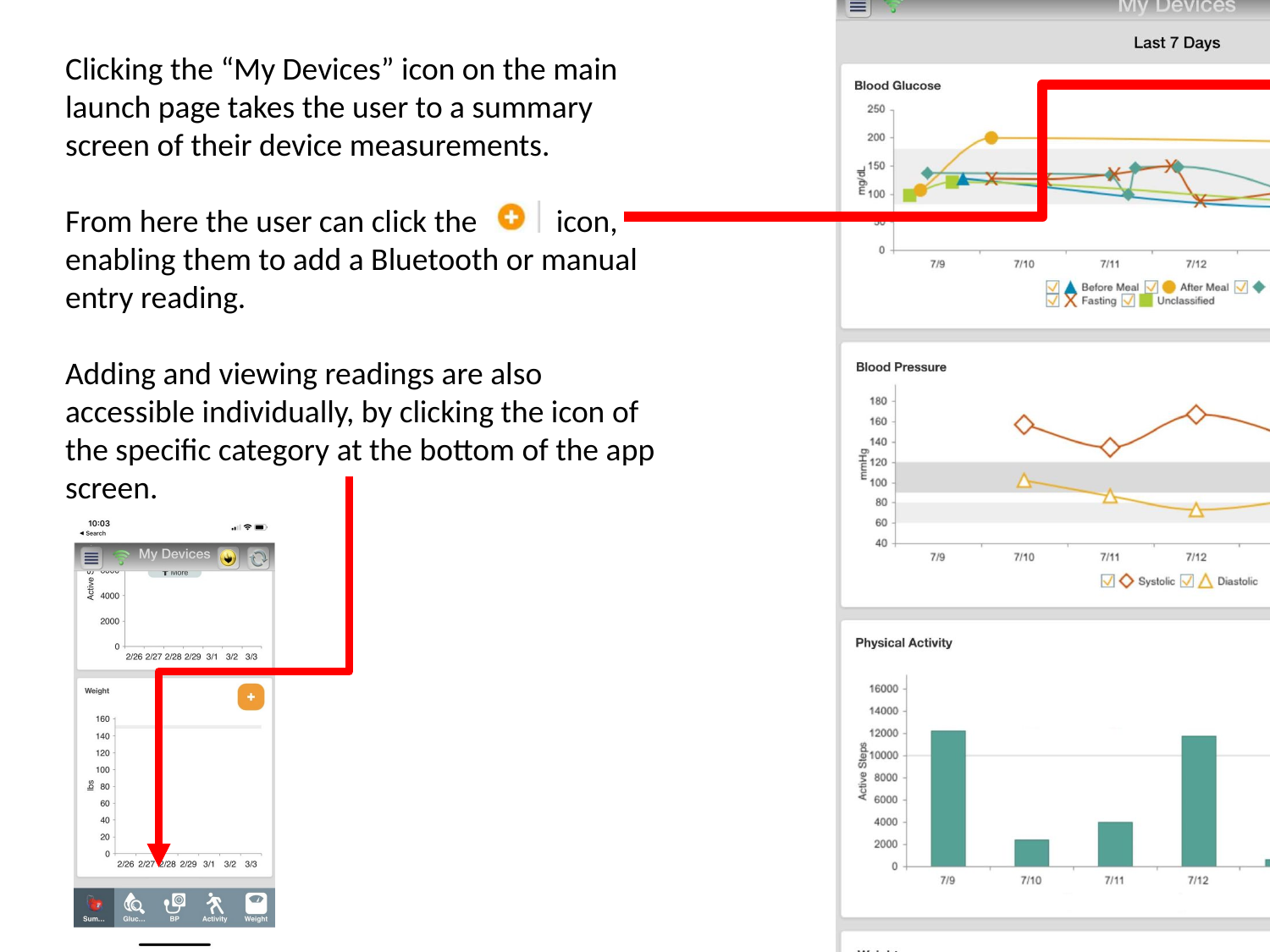

Clicking the “My Devices” icon on the main launch page takes the user to a summary screen of their device measurements.
From here the user can click the icon, enabling them to add a Bluetooth or manual entry reading.
Adding and viewing readings are also accessible individually, by clicking the icon of the specific category at the bottom of the app screen.

## Slide 6
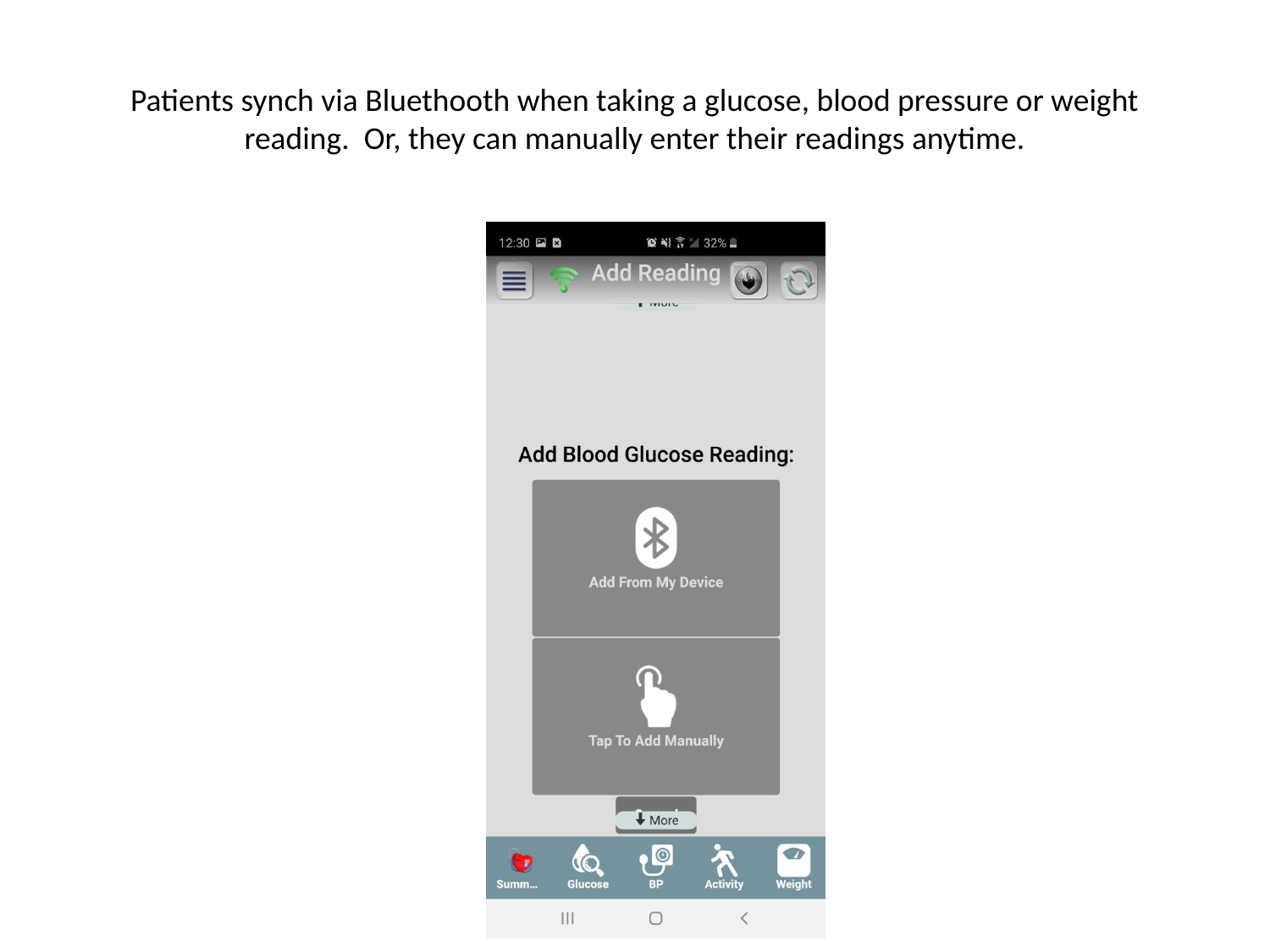

# Patients synch via Bluethooth when taking a glucose, blood pressure or weight reading. Or, they can manually enter their readings anytime.

## Slide 7
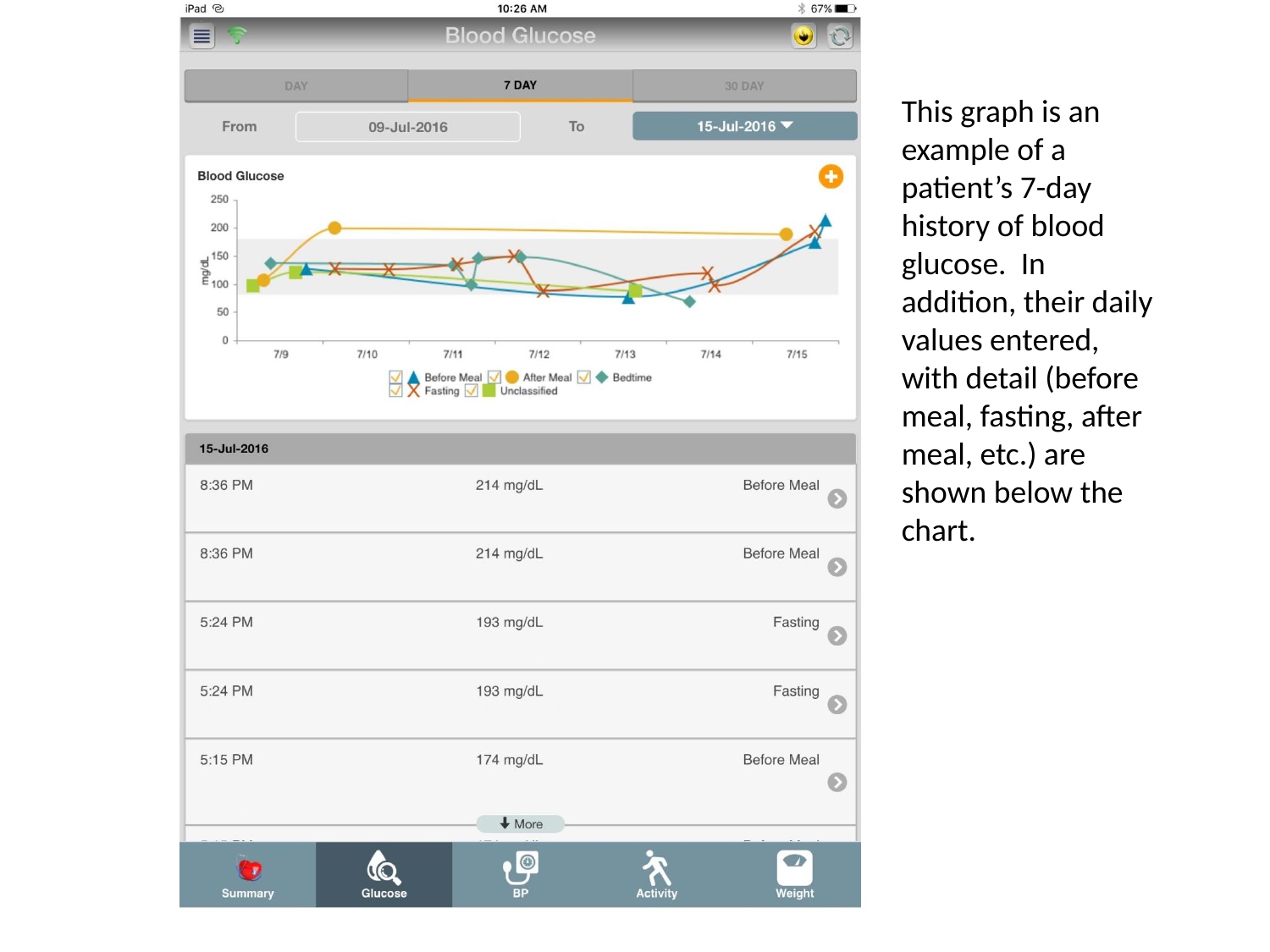

This graph is an example of a patient’s 7-day history of blood glucose. In addition, their daily values entered, with detail (before meal, fasting, after meal, etc.) are shown below the chart.

## Slide 8
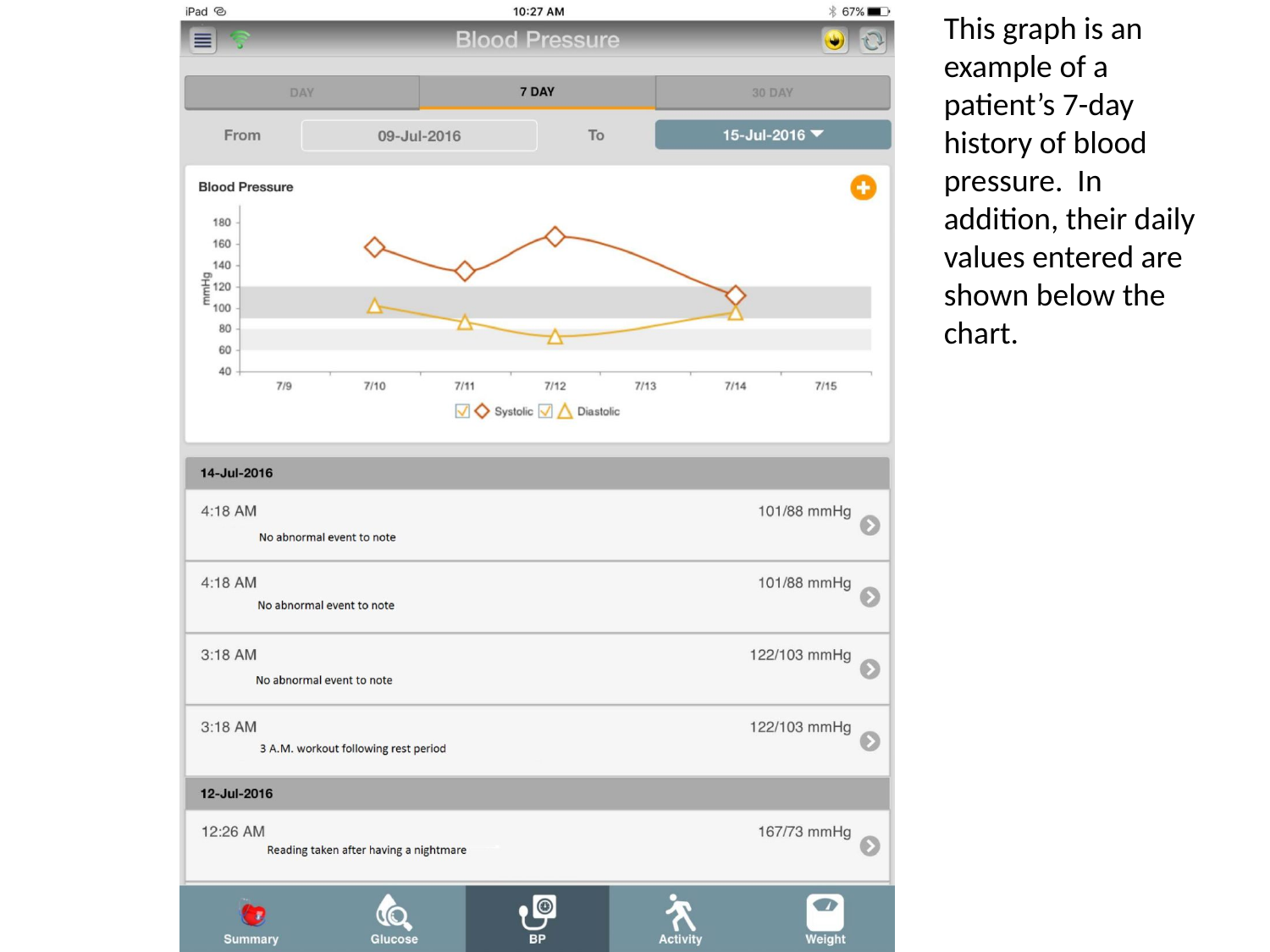

This graph is an example of a patient’s 7-day history of blood pressure. In addition, their daily values entered are shown below the chart.

## Slide 9
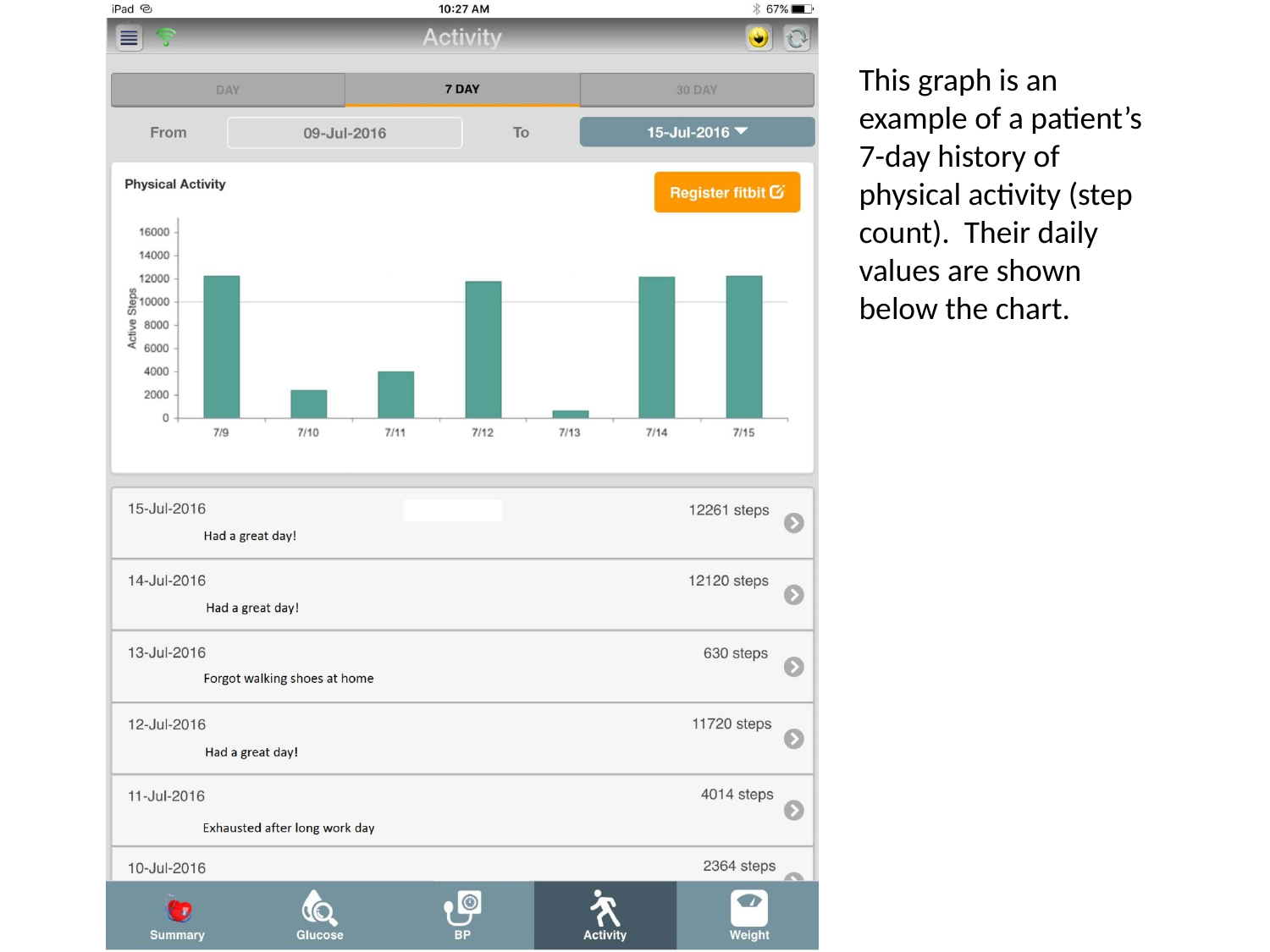

This graph is an example of a patient’s 7-day history of physical activity (step count). Their daily values are shown below the chart.

## Slide 10
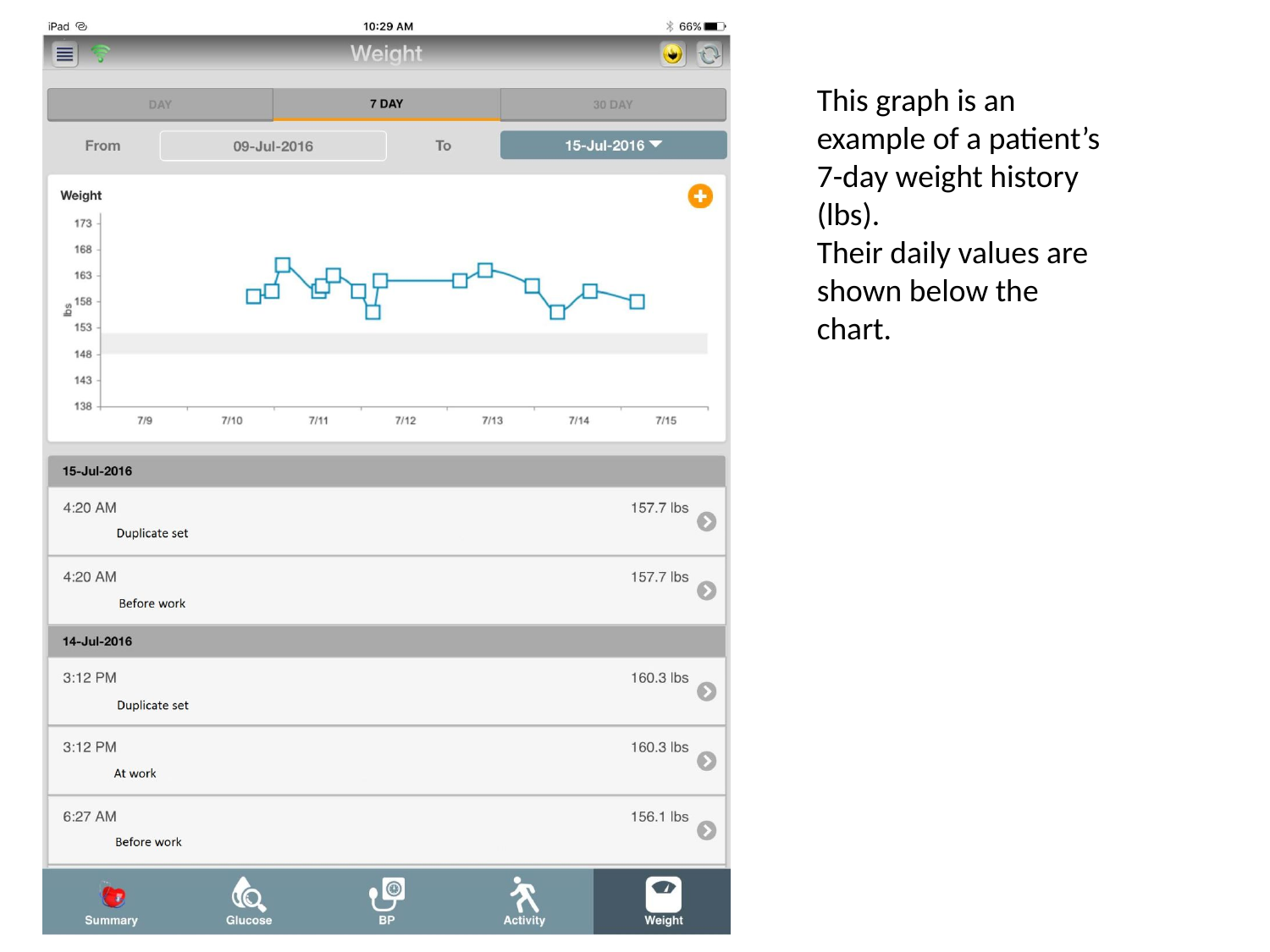

This graph is an example of a patient’s
7-day weight history (lbs).
Their daily values are shown below the chart.
