# Supplementary material for: Enhancing Patient Activation and Self-Management Activities in Patients With Type 2 Diabetes Using the US Department of Defense Mobile Health Care Environment: Feasibility Study
Source: J Med Internet Res. 2020 May 26;22(5):e17968. doi: 10.2196/17968 (PMC7284404; doi:10.2196/17968)
Supplement: Multimedia Appendix 2 [file jmir_v22i5e17968_app2.pptx]

## Slide 1
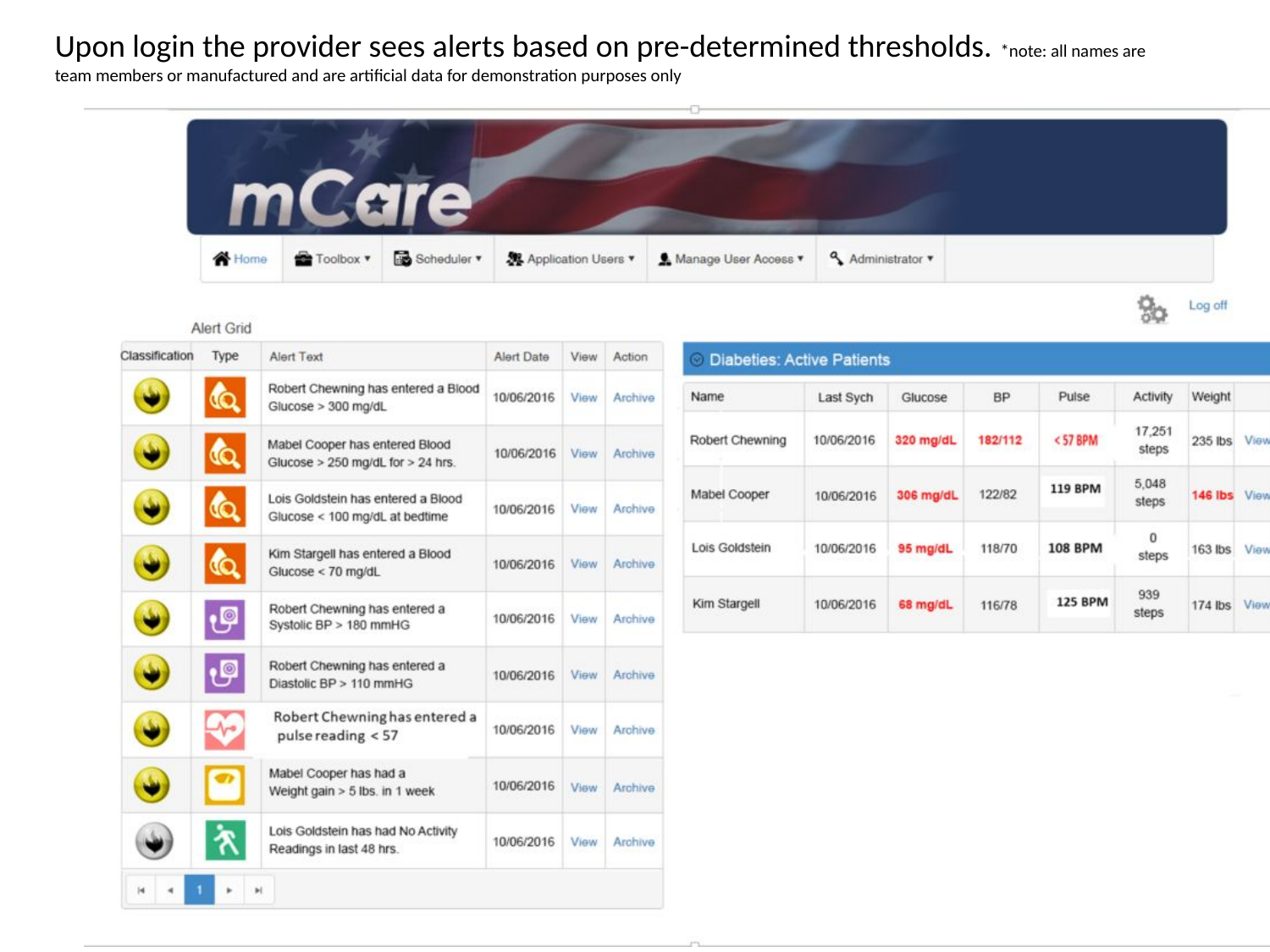

Upon login the provider sees alerts based on pre-determined thresholds. *note: all names are team members or manufactured and are artificial data for demonstration purposes only

## Slide 2
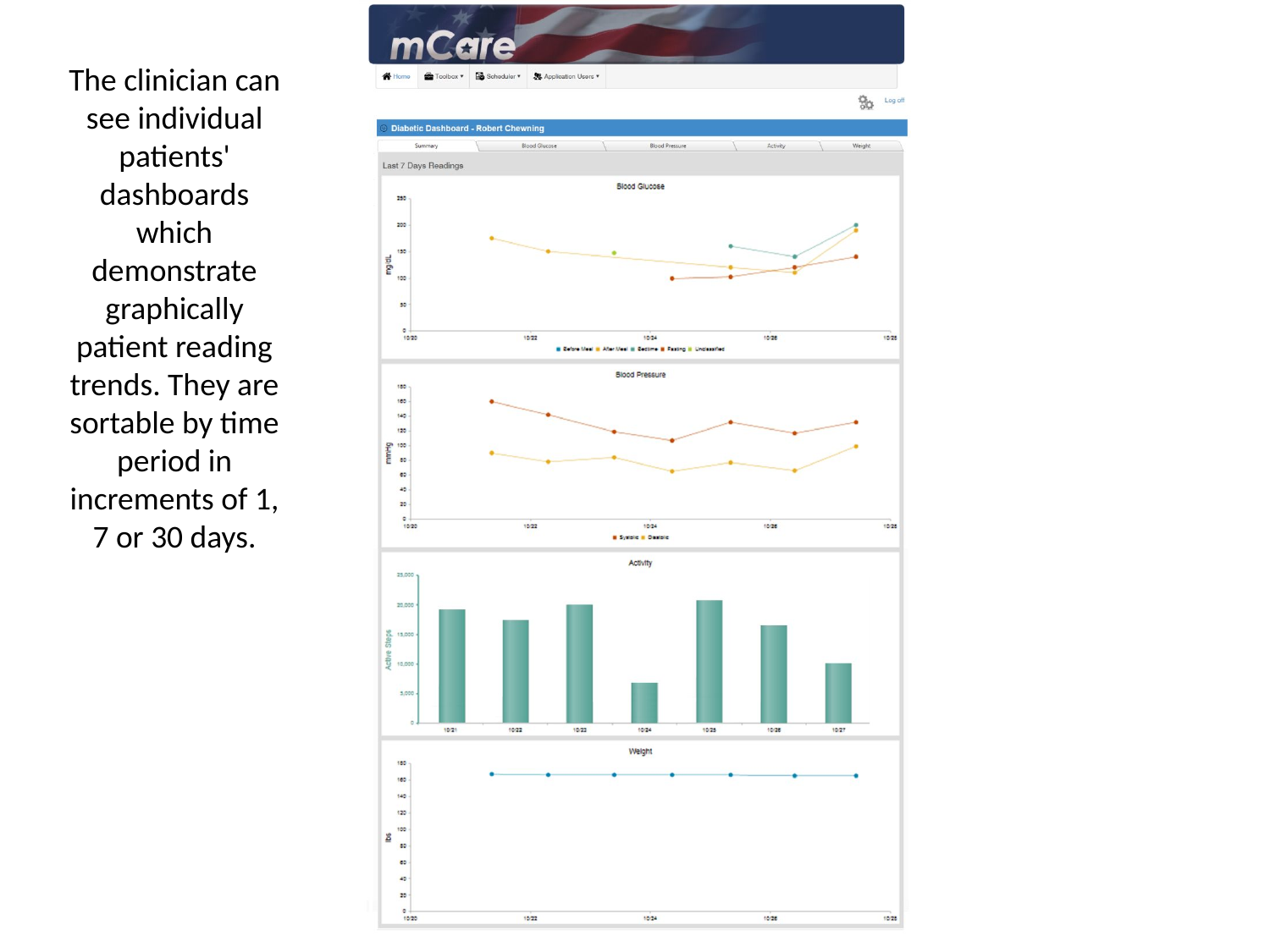

The clinician can see individual patients' dashboards which demonstrate graphically patient reading trends. They are sortable by time period in increments of 1, 7 or 30 days.

## Slide 3
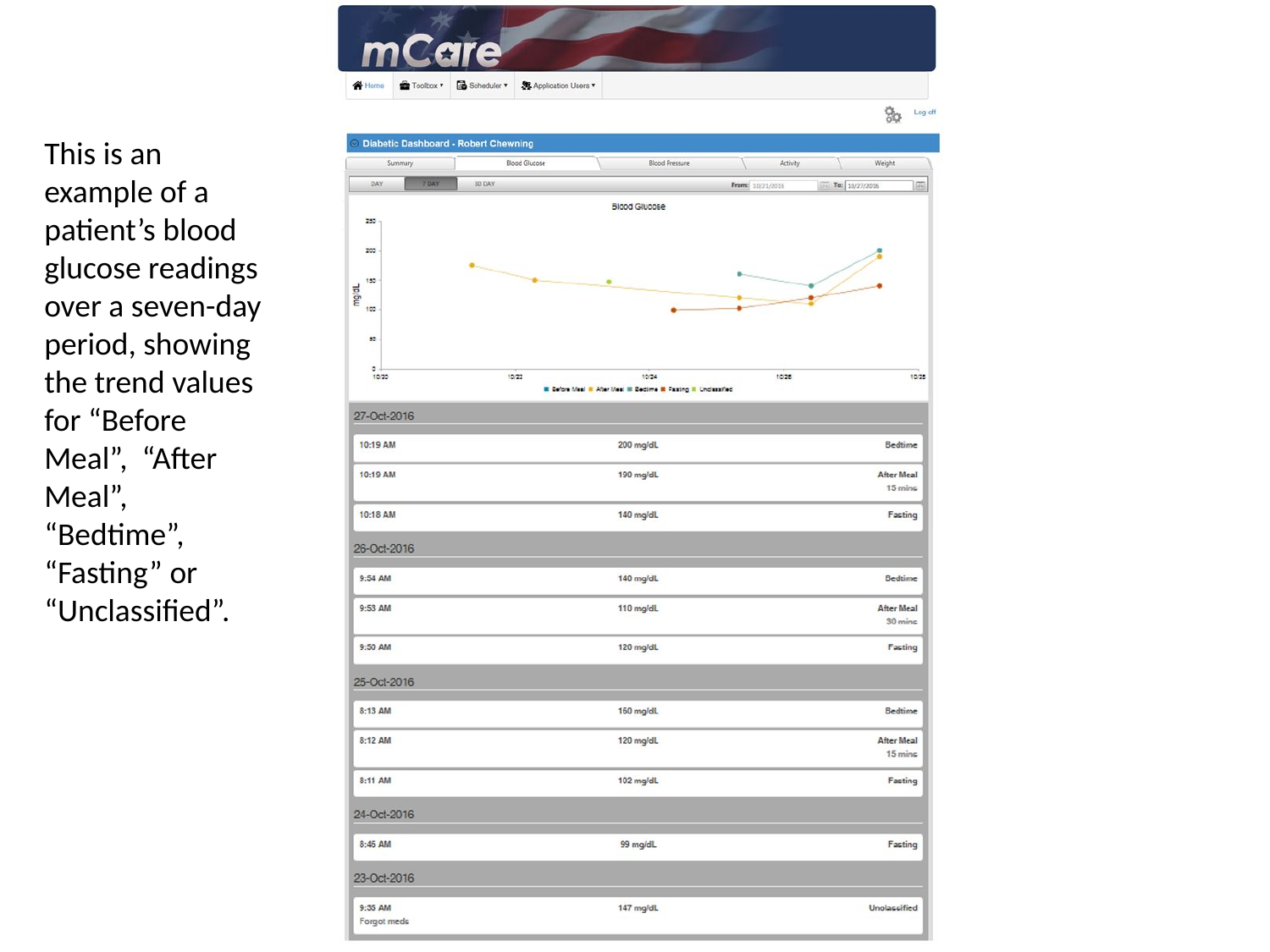

This is an example of a patient’s blood glucose readings over a seven-day period, showing the trend values for “Before Meal”, “After Meal”, “Bedtime”, “Fasting” or “Unclassified”.

## Slide 4
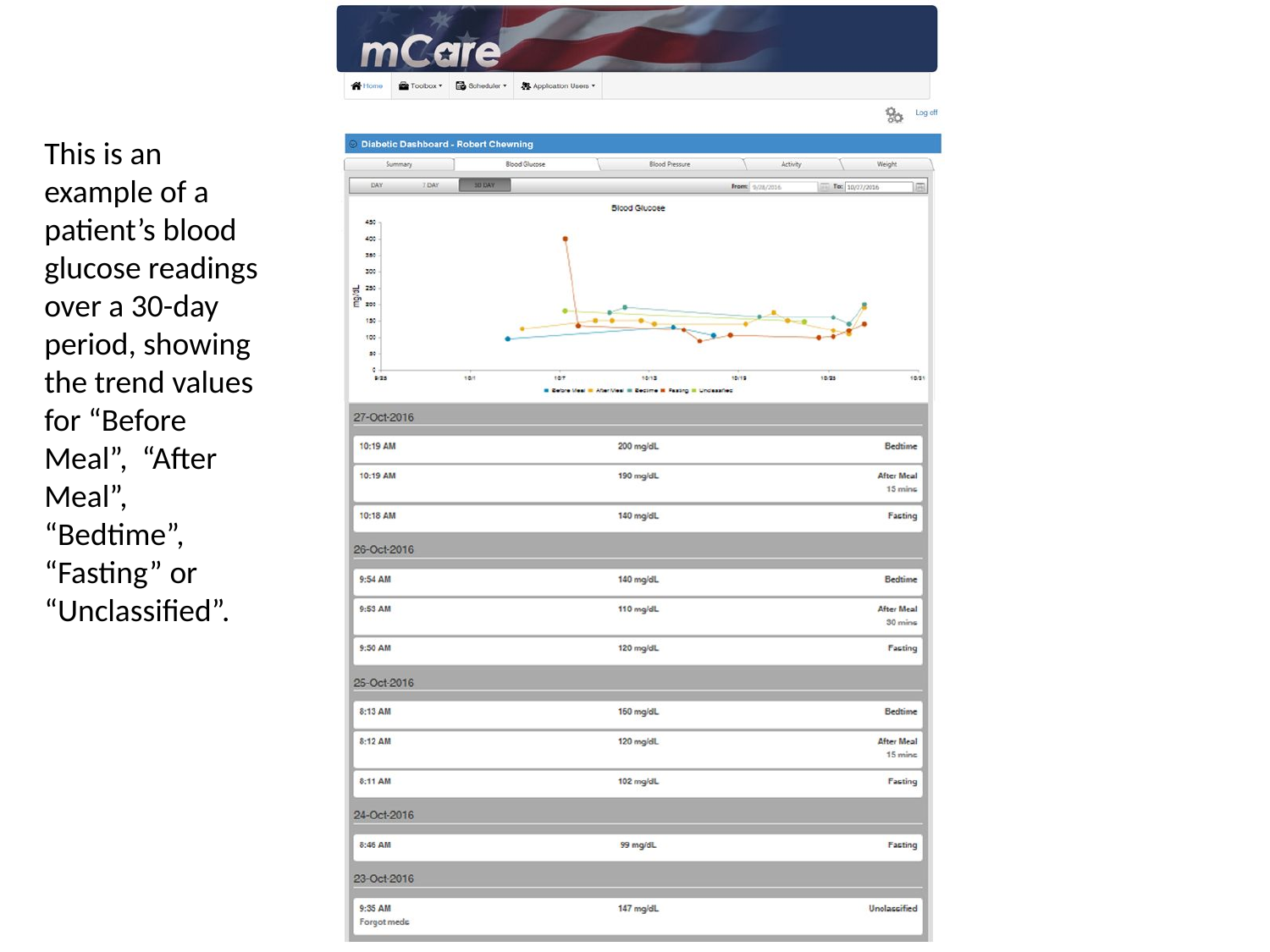

This is an example of a patient’s blood glucose readings over a 30-day period, showing the trend values for “Before Meal”, “After Meal”, “Bedtime”, “Fasting” or “Unclassified”.

## Slide 5
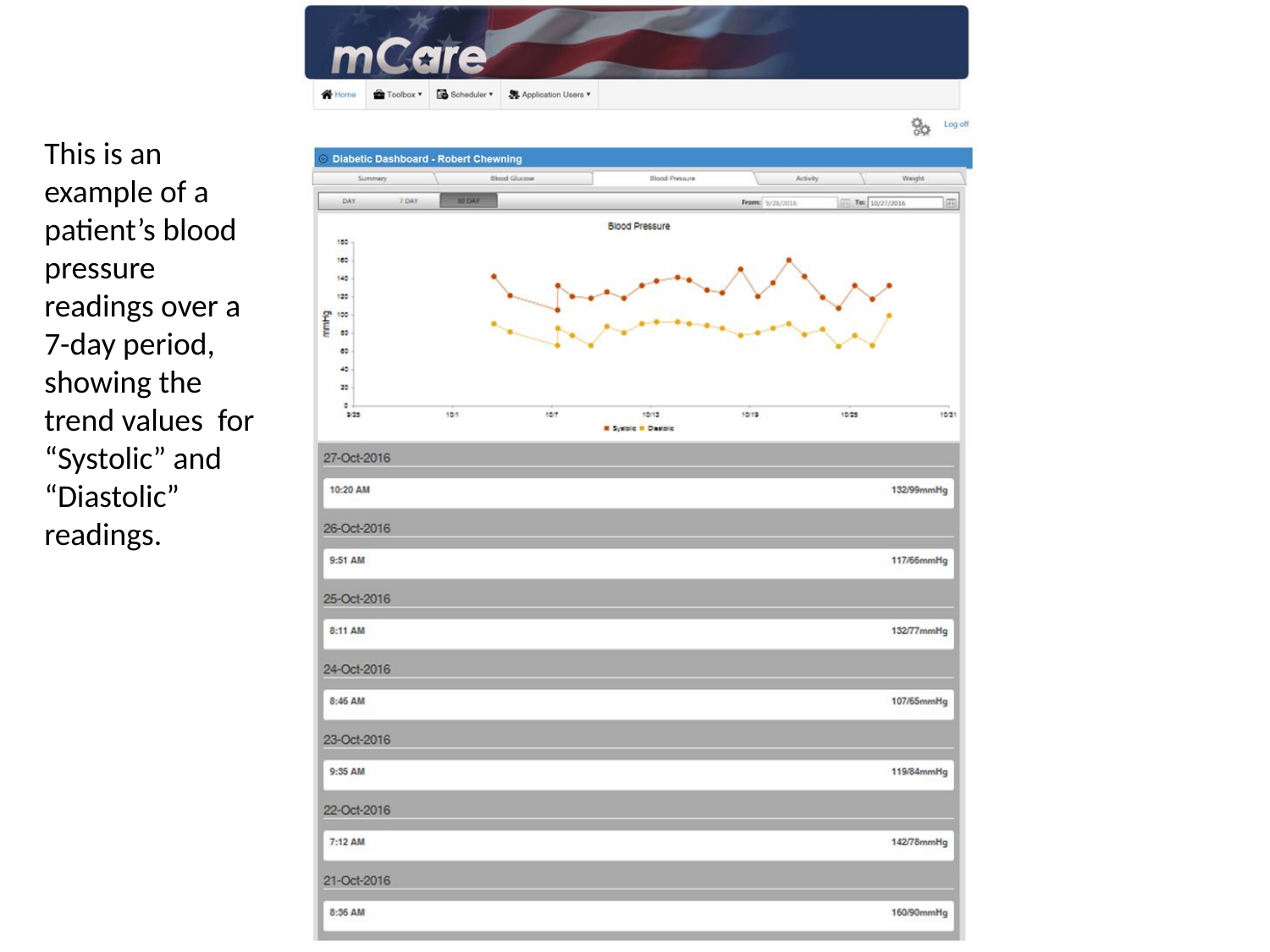

This is an example of a patient’s blood pressure readings over a 7-day period, showing the trend values for “Systolic” and “Diastolic” readings.

## Slide 6
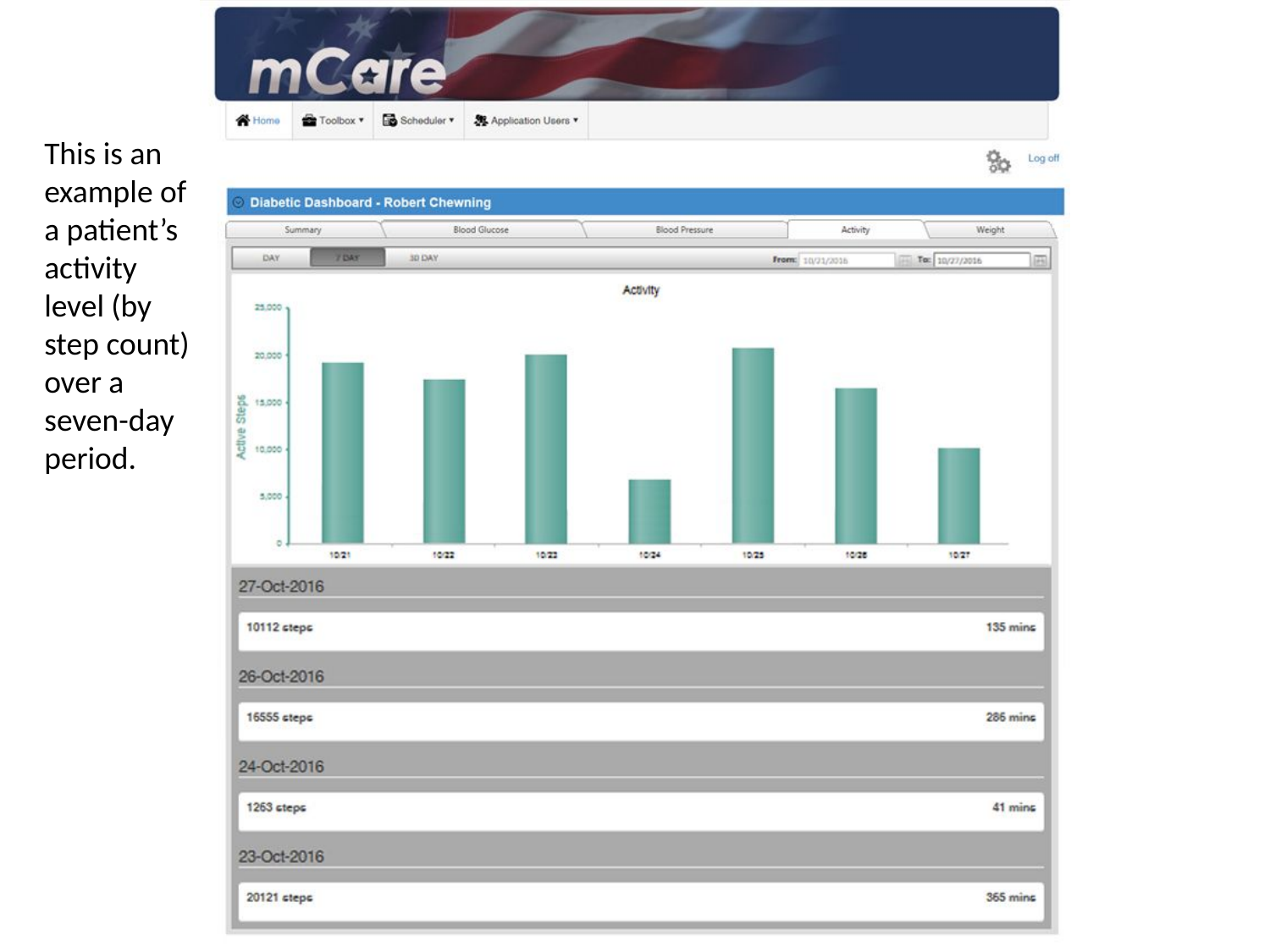

This is an example of a patient’s activity level (by step count) over a seven-day period.

## Slide 7
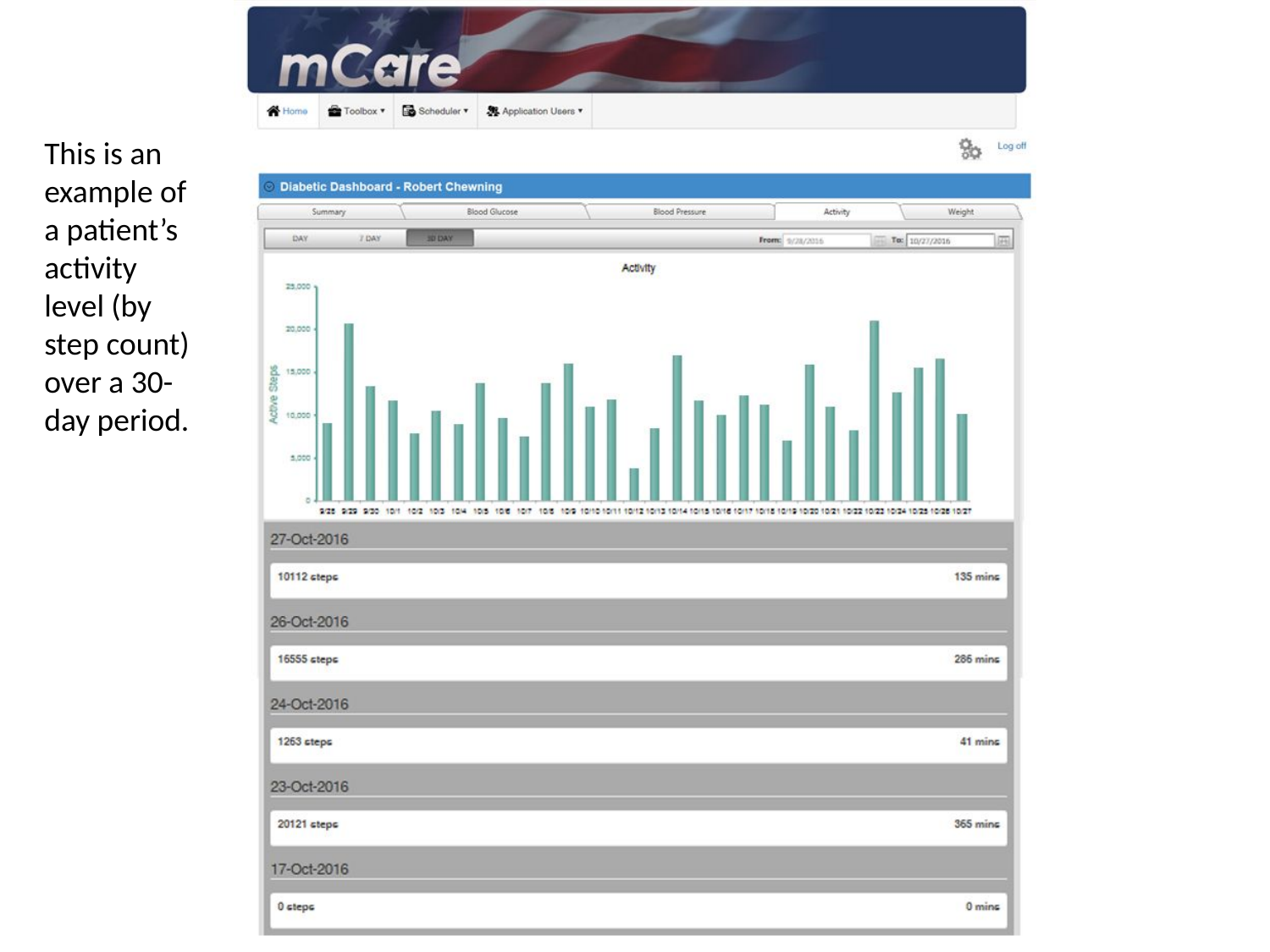

This is an example of a patient’s activity level (by step count) over a 30-day period.

## Slide 8
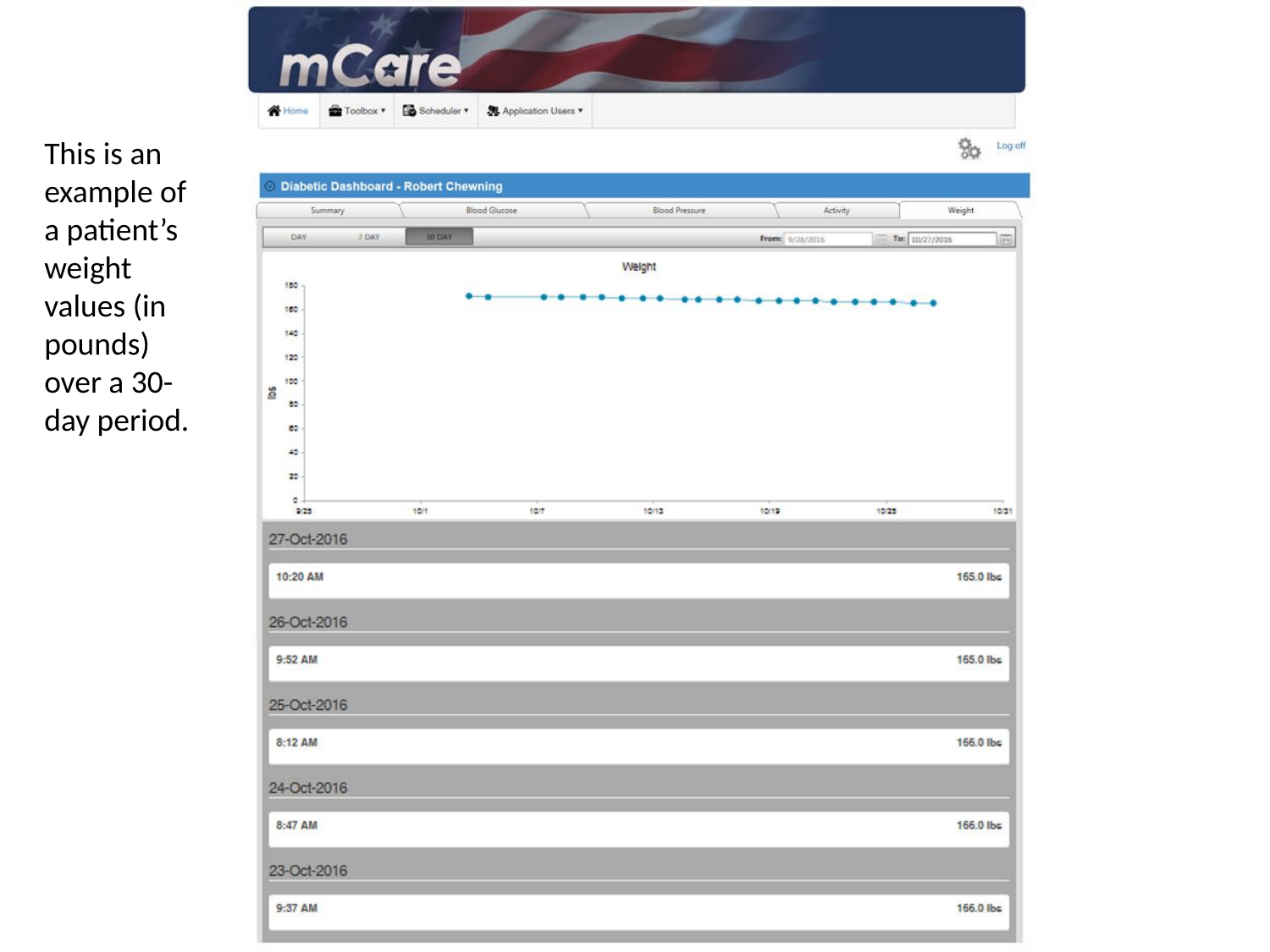

This is an example of a patient’s weight values (in pounds) over a 30-day period.

## Slide 9
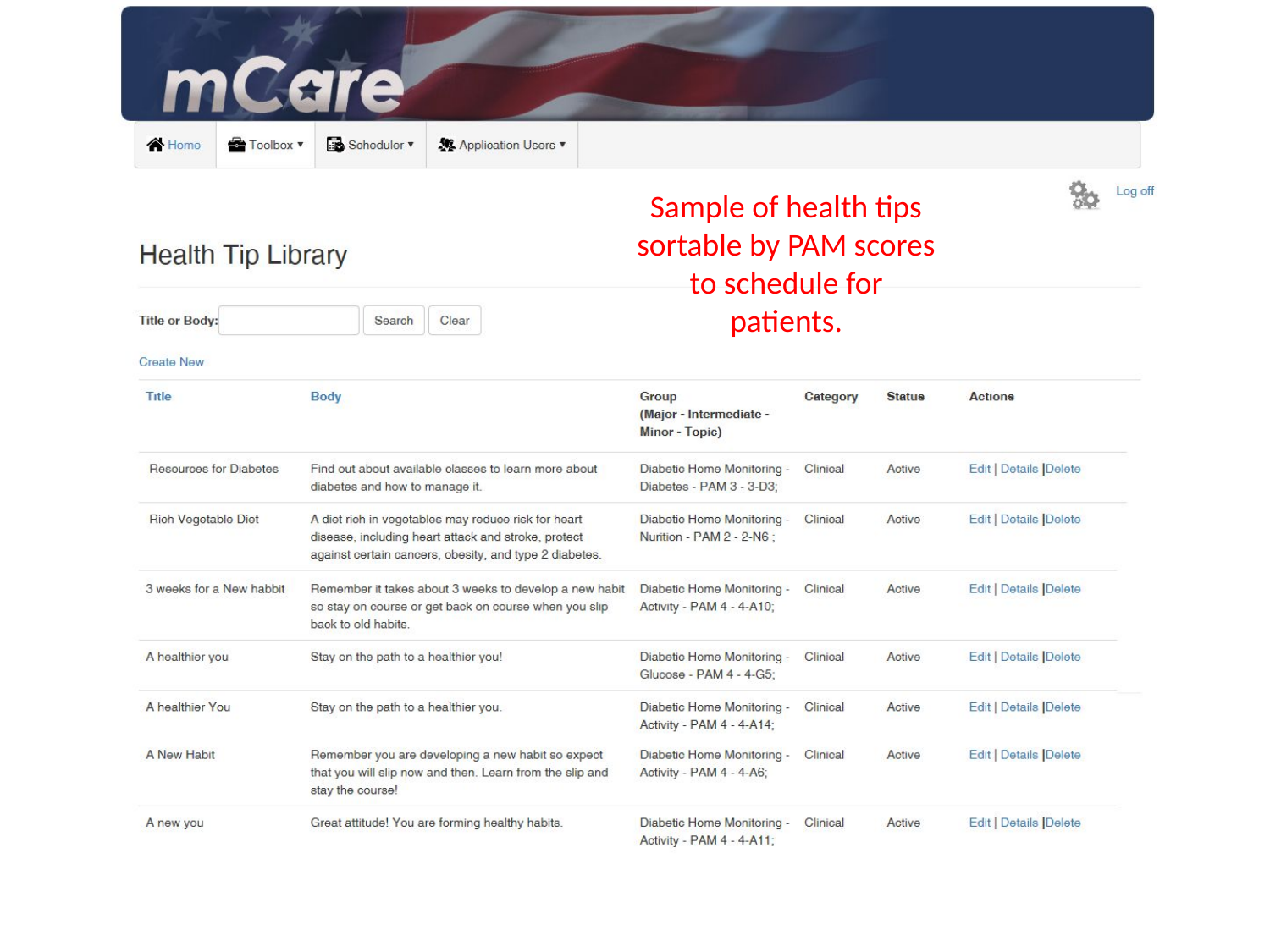

Sample of health tips sortable by PAM scores to schedule for patients.
