# Supplementary material for: Enhancing Patient Activation and Self-Management Activities in Patients With Type 2 Diabetes Using the US Department of Defense Mobile Health Care Environment: Feasibility Study
Source: J Med Internet Res. 2020 May 26;22(5):e17968. doi: 10.2196/17968 (PMC7284404; doi:10.2196/17968)
Supplement: Multimedia Appendix 3 [file jmir_v22i5e17968_app3.pdf]

Enhancing mHealth Technology in the PCMH Environment to Activate Chronic Care Patients

VISIT Phase 1 Data Collecting Sheet

DEMOGRAPHY

Subject# :________________________ **Visit Date:** ___________________ **Visit Time:** __________________

First Name:____

Introductory Question

In general, how comfortable do you feel with using mobile apps or technology?

**Very comfortable**	**Comfortable**	**Neutral**	**Uncomfortable**	**Very Uncomfortable**
☐	☐	☐	☐	☐

Usability tasks

**Task**	**Task completion coding**	**Notes (error coding)**
Locate mCare icon		
Log in		
Accept Disclaimer		
Understand Disclaimer		
Locate Announcements or Health Tip Icon		
Navigate to Health Tips		
Navigate to Settings		

Setting Goals

	**Single Ease (1-7)**	**Task Completion Coding**	**Error Coding**	**Efficiency (Time)**
Set a **BP goal** (low and high for both systolic and diastolic)				
Set a **glucose** goal (low and high)				
Set a **weight** goal				
Set a **steps** per day goal				

Application Satisfaction Questions

Is there anything confusing or that you don't understand regarding the tasks up to this point?
***Yes*☐ *No* ☐**
If yes, what is confusing and why?

Is there anything about how the application looks (for instance the format of the layout of the application) that you would change? *Yes*☐ *No*☐
If yes, what would you change and why?

From what you have seen so far, is there anything about how the application works that you would change?
***Yes*☐ *No* ☐**
If yes, what would you change and why?

Blood Pressure (BP) Monitoring Component

	**Single Ease (1-7)**	**Task Completion Coding**	**Error Coding**	**Efficiency (Time)**
Add Blood Pressure Manually of 132/85				
Add Blood Pressure Bluetooth				

Question 1: Which day last week was the systolic blood pressure (the top number) the highest?
***Answered correctly? Yes*☐ *No*☐**

BP Application Satisfaction Questions

Is there anything in the BP portion that is confusing or that you don't understand? *Yes*☐ *No* ☐
If yes, what part is confusing and why?

Is there anything about how the BP portion looks (for instance the format of the layout of the application) that you would change? *Yes*☐ *No*☐
If yes, what would you change and why?

Is there anything about how the BP portion works that you would change? *Yes*☐ *No* ☐
If yes, what would you change and why?

Glucose Monitoring Component				
	**Single Ease (1-7)**	**Task Completion Coding**	**Error Coding**	**Efficiency (Time)**
Add Glucose Manually of 400				

Question 1: Can you locate the safety alert? ***Yes*** ☐ ***No*** ☐

Question 2: Can you navigate to the safety alert? ***Yes*** ☐ ***No*** ☐

Question 3: What is the alert asking you to do?
Interpreted correctly? Yes ☐ ***No*** ☐

Question 4: What was the fasting blood sugar reading yesterday?
Answered correctly? Yes ☐ ***No*** ☐

Question 5: During the past week, were there any times that the blood sugar was higher than the target range?
Answered correctly? Yes ☐ ***No*** ☐

Question 6: What was the average bedtime glucose reading from (insert 4 days period)?
Answered correctly? Yes ☐ ***No*** ☐

Glucose Application Satisfaction Questions

Is there anything in the glucose portion that is confusing or that you don't understand? ***Yes*** ☐ ***No*** ☐
If yes, what is confusing and why?

Is there anything about how the glucose application looks (for instance the format or the layout of the application) that you would change? ***Yes*** ☐ ***No*** ☐
If yes, what would you change and why?

Is there anything about how the glucose portion works that you would change? ***Yes*** ☐ ***No*** ☐
If yes, what would you change and why?

Weight/Scale Monitoring Component

	Single Ease (1-7)	Task Completion Coding	Error Coding	Efficiency (Time)
Add Weight Manually of 175				
Add Weight Bluetooth				

Question 1: What was the lowest weight recorded for the past week?
***Answered correctly? Yes*☐ *No*☐**

Question 2: Has the weight increased, decreased or stayed the same in the past month?
***Answered correctly? Yes*☐ *No*☐**

Weight/Scale Application Satisfaction Questions

Is there anything in the weight/scale portion that is confusing or that you don't understand? *Yes*☐ *No* ☐
If yes, what is confusing and why?

Is there anything about how the weight/scale portion looks (for instance the format of the layout of the application) that you would change? *Yes*☐ *No*☐
If yes, what would you change and why?

Is there anything about how the scale/weight portion works that you would change? *Yes*☐ *No* ☐
If yes, what would you change and why?

Activity Monitoring Component

Question 1: Which date last week had the greatest number of activity minutes?
***Answered correctly? Yes*☐ *No*☐**

Question 2: What is the total number of steps taken last week?
***Answered correctly? Yes*☐ *No*☐**

Question 3: This device will track steps and active minutes. Which would you personally use to track your activity? Steps? ☐ Active minutes? ☐ Both? ☐

**Activity Application Satisfaction Questions**
**Is there anything in the activity portion that is confusing or that you don't understand? *Yes*☐ *No* ☐**<br>**If yes, what is confusing and why?**
**Is there anything about how the activity portion looks (for instance the format of the layout of the application) that you would change? *Yes*☐ *No*☐**<br>**If yes, what would you change and why?**
**Is there anything about how the activity application works that you would change? *Yes*☐ *No* ☐**<br>**If yes, what would you change and why?**
**Additional Questions**
**If available, would you manually enter dated past readings for glucose, blood pressure or weight? *Yes*☐ *No* ☐**
