# Supplementary material for: Enhancing Patient Activation and Self-Management Activities in Patients With Type 2 Diabetes Using the US Department of Defense Mobile Health Care Environment: Feasibility Study
Source: J Med Internet Res. 2020 May 26;22(5):e17968. doi: 10.2196/17968 (PMC7284404; doi:10.2196/17968)
Supplement: Multimedia Appendix 4 [file jmir_v22i5e17968_app4.pdf]

Clinician Focus Groups – Guide for facilitators

After previewing the features of the mCare system, our first set of questions for you today are about specific components/features of the system. We want you to share your thoughts on ease of use for the patients, how mCare may impact treatment effectiveness and patient satisfaction. We want you to share your preferences and concerns.

1. Please tell us your initial impressions of the design of the MHCE interface in this current version. In doing so, please distinguish between "concerns" vs. "preferences".

[Facilitator Note: Pause]

[Facilitator Note: If not addressed in their initial response use specific probes below]

What are your general thoughts about the Homepage screen, Blood pressure component, glucose component, weight component and physical activity component? .

Ease of use for patients?

How will this have an impact (or influence) on treatment effectiveness?

Your satisfaction with these components?

Your thoughts on how this will influence patient satisfaction?

Thank you, next we would like to talk about the **alerts** provided through MHCE.

We are going to begin by showing you the algorithms developed to deliver an alert to patients based on threshold clinical values and would like your feedback on the accuracy and appropriateness of the alerts.

SHOW SLIDE SET OF SAFETY ALERTS

2. Patients will go through the following steps to get the alert.[*Facilitator Note: walk through the steps*] what is your overall impression of the built in alerts ?
[*Facilitator Note: Pause*]

2.a What do you think of the wording of the alert text messages?

How appropriate is the language with regard to patient safety?

If concerns, please explain

2.b What are your thoughts on the ease of understanding for the alert messages?

2.c What are your thoughts on alert messages ability to influence patient to take action, if needed?

Is the wording of the safety alert text messages appropriate for patient safety and for them to take action, if needed? If not, please explain.

Is the wording of the safety alert text messages easy for a patient to comprehend? If not, please explain.

A **back-end portal** of the MHCE system where patient information is summarized will also be available to clinicians.

3. With respect to a "back end portal" (where the provider can view the data from the patients Bluetooth devices), what information would be most helpful to you?
   [*Facilitator Note: Pause*]

   [Facilitator Note: If not mentioned in response to previous question probe on the following list of potential content for "back end portal"]

   3.a How about inclusion of the following:
   - Glucose history
   - BP history
   - Weight History
   - Step Count History (Preference of steps vs active minutes)

   3.b For each type of information you would like to have included, what is the best summary format for the information you would like to have available through the "back end portal"?

   [*Facilitator Note: Show options (what are these options? Monthly, weekly, etc.?*]

   - Glucose history
   - BP history
   - Weight History
   - Step Count History

The next questions are in regards to how this system and the backend portal will impact workflow in your work setting.

4.a Who is most likely to use a back end portal summary (such as nurses, providers, others)?

4.b How do you see yourself using this MHCE system with patients?

4.c How could this positively or negatively impact workflow?

4.d How could this positively or negatively impact the patient encounter?
