# Supplementary material for: Enhancing Patient Activation and Self-Management Activities in Patients With Type 2 Diabetes Using the US Department of Defense Mobile Health Care Environment: Feasibility Study
Source: J Med Internet Res. 2020 May 26;22(5):e17968. doi: 10.2196/17968 (PMC7284404; doi:10.2196/17968)
Supplement: Multimedia Appendix 5 [file jmir_v22i5e17968_app5.pdf]

Exploring the Usability of a Mobile Application for Improving Chronic Disease Self-Management in the Patient Centered Medical Home Environment

DISCLAIMER

This material is based upon work supported by the U.S. Army Medical Research Acquisition Activity under Contract No. W81XWH-15-C-0070. Any opinions, findings and conclusions or recommendations expressed in this material are those of the author(s) and do not necessarily reflect the views of the U.S. Army Medical Research Acquisition Activity.

Presentation Outline

- Provide an overview of larger study
- Describe Phase 1 usability testing:
  - Background
  - Objectives
  - Methods
  - Findings

Research Group Collaborators

- Government, Academia, Industry partnership
  - Development Advisory Team
    - Military Health, University, IMS Health
  - Clinical Advisory Team
  - Intervention Team
  - Data Analysis Team

Background – Research Study

- Multi-site Military Health System study
  - U.S. Department of Defense W81XWH-15-C-0070 Gimbel (PI) “Enhancing mHealth Technology in the PCMH Environment to Activate Chronic Care Patients” 08/15 – 01/18
    - Mike O'Callaghan Federal Medical Center (NV)
    - Madigan Army Medical Center (WA)

- Phase 1: Utilized a participatory approach in guiding adaptations of a health system application, called mCare.

- Phase 2: Feasibility study where 120 patients are randomized to biosensors only vs. biosensors + mCare interface + tailored messaging

- Gimbel et al. JMIR Res Protoc 2017;6(3):e38. DOI: 10.2196/resprot.6993

Background – Biosensors & mCare

- Glucometer: My GlucoHealth

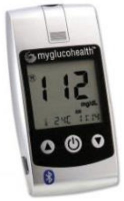

- Blood Pressure Monitor: A&D

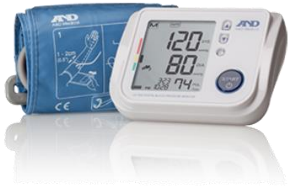

- Weight Scale: A&D

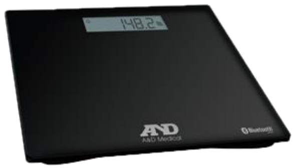

- Activity Monitor:
  - Fitbit Charge (wrist)

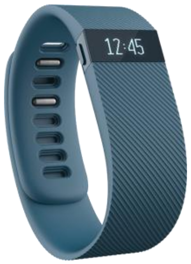

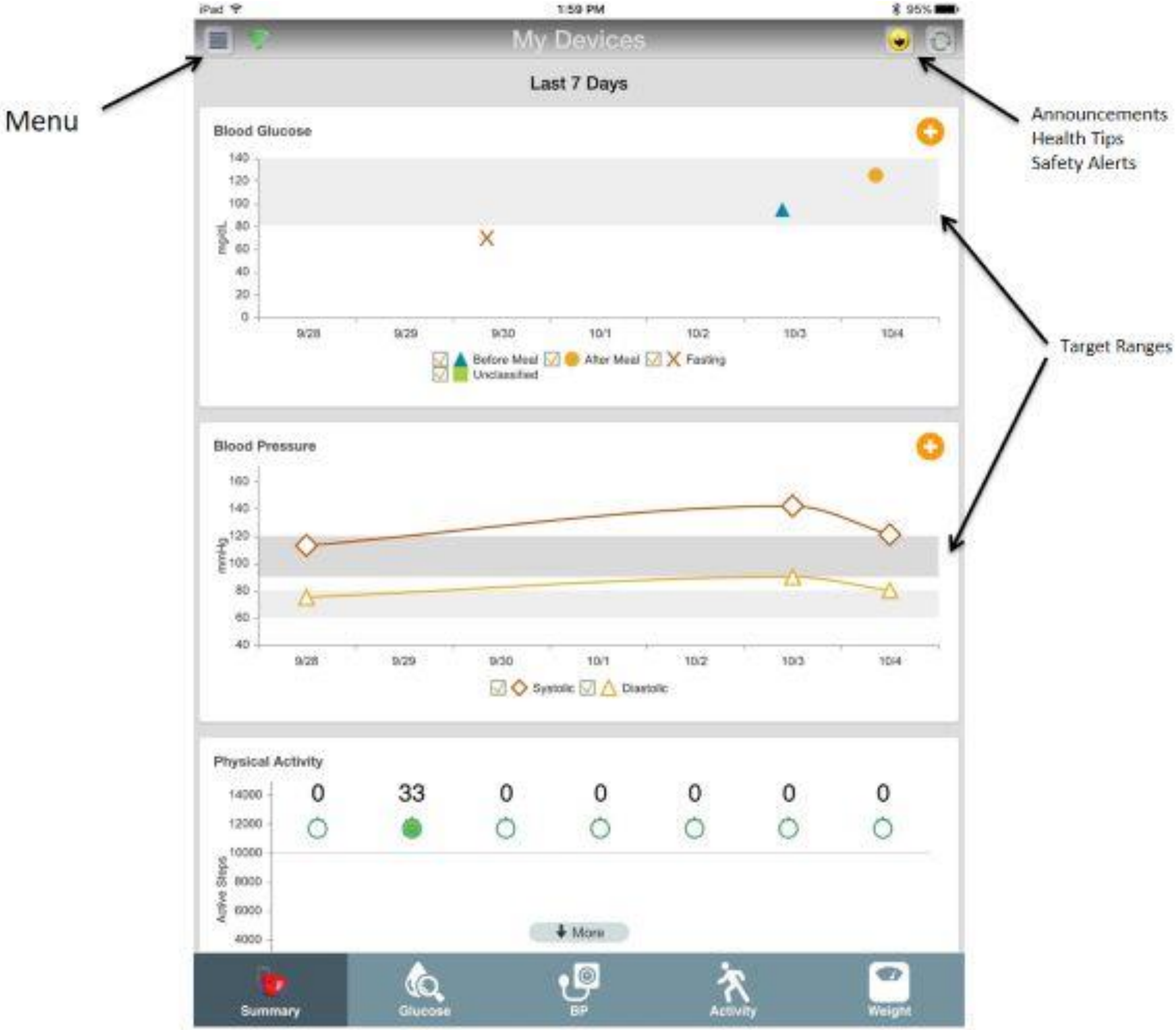

iPad
1:59 PM
95%
My Devices
Last 7 Days
Menu
Announcements
Health Tips
Safety Alerts
Target Ranges
Blood Glucose
mg/dL
140
120
100
80
60
40
20
0
9/28
9/29
9/30
10/1
10/2
10/3
10/4
Before Meal
After Meal
Fasting
Unclassified
Blood Pressure
mmHg
160
140
120
100
80
60
40
9/28
9/29
9/30
10/1
10/2
10/3
10/4
Systolic
Diastolic
Physical Activity
Active Steps
14000
12000
10000
8000
6000
4000
0
33
0
0
0
0
0
More
Summary
Glucose
BP
Activity
Weight

mCare

Home | Toolbox ▾ | Scheduler ▾ | Application Users ▾ | Manage User Access ▾ | Administrator ▾

Log off

Alert Grid

Alert URL	Alert Text	Alert Date	View	Action
	Robert Chewning has entered a Blood Glucose > 300 mg/dL	10/06/2016	View	Archive
	Mabel Cooper has entered Blood Glucose > 250 mg/dL for > 24 hrs.	10/06/2016	View	Archive
	Lois Goldstein has entered a Blood Glucose < 100 mg/dL at bedtime	10/06/2016	View	Archive
	Kim Stargell has entered a Blood Glucose < 70 mg/dL	10/06/2016	View	Archive
	Robert Chewning has entered a Systolic BP > 180 mmHG	10/06/2016	View	Archive
	Robert Chewning has entered a Diastolic BP > 110 mmHG	10/06/2016	View	Archive
	Mabel Cooper has had a Weight gain > 5 lbs. in 1 week	10/06/2016	View	Archive
	Lois Goldstein has had No Activity Readings in last 48 hrs.	10/06/2016	View	Archive

1

Diabeties: Active Patients

Name	Last Sych	Glucose	BP	Activity	Weight	
Robert Chewning	10/06/2016	**320 mg/dL**	**182/112**	17,251 steps	235 lbs	View
Mabel Cooper	10/06/2016	**306 mg/dL**	122/82	5,048 steps	**146 lbs**	View
Lois Goldstein	10/06/2016	**95 mg/dL**	118/70	**0 steps**	163 lbs	View
Kim Stargell	10/06/2016	**68 mg/dL**	116/78	939 steps	174 lbs	View

Phase 1 Objective & Methods

- Utilize a participatory approach to engage Type 2 diabetics, and the clinicians who treat them, in guiding adaptations of the mCare interface to be used in Phase 2 of this study.

- Mixed methods approach:

<u>Quantitative</u>
- Patient task observations
- Open-ended questions during usability testing to capture Patient thoughts

<u>Quantitative</u>
- Patient + Clinician feedback
- Patient task completion notes

Methods – Design

- Science Panel on Interactive Communication and Health (Robinson et al., 1998)
- International Organization for Standardization (ISO) 9241-11 Usability framework : usability definitions and evaluation metrics
- Georgsson & Staggers (2016) model
- Mixed methods evaluation (QUAN + QUAL)

Methods – Context of Use (Patients)

- **Users:** Describe relevant characteristics of the users.
- **Tasks:** Specific activities (device interactions) that will be required of users.
- **Equipment:** Hardware, software and peripheral devices (My GlucoHealth glucometer, A&D blood pressure monitor, A&D weight scale, Fitbit Charge).
- **Environment:** Relevant characteristics of the testing environment including: technical (WiFi), and physical (testing space).

Methods – Usability Metrics (Patients)

- **Effectiveness:** Extent to which the user can achieve a goal with accuracy and completeness.
  - *(1) the degree of task completion AND*
  - *(2) total number of errors per task*

- **Efficiency:** Level of effort and resource usage which is required by the user in order to achieve a goal in relation to accuracy and completeness.
  - *Timed each individual task and compute average time for each task across users.*

- **Satisfaction:** Extent to which users are free from discomfort, and their attitudes towards the use of the product.
  - *Single Ease Question (SEQ – Sauro & Dumas, 2009)*
  - *Open-ended questions (developed by Phase 1 Team)*
  - *System Usability Scale (SUS – Sauro & Lewis, 2012)*

Methods – Usability Tasks (Patients)

- Login and System navigation
- Goal setting
- Specific tasks for each biosensor:
  - Blood pressure (manual entry, Bluetooth sync, graphs)
  - Blood sugar (manual entry, graphs)
  - Weight scale (manual entry, Bluetooth sync, graphs)
  - Fitbit (graph interpretation only)

Methods – Usability (Clinicians)

- Focus groups
  - Physicians and Clinical Staff
    - Recorded
    - Field notes
- Feedback on:
  - Perceived usability for patient
  - Workflow issues / concerns
  - Message content - system alerts, patient reminders
  - Backend portal (doctor's view)

Findings – Patients

- All diabetics indicated that mCare would “help them manage their diabetes” and give their healthcare provider a “better report of their health.” As expected, patients rated navigation tasks as less difficult and peripheral device tasks, e.g., syncing, as more difficult.

- Diabetics committed fewer errors with basic navigation tasks and more with peripheral devices. Further, a higher proportion of diabetics made errors with external device tasks.

- Diabetics and clinicians alike suggested minor changes regarding the look and function of the application, e.g., adding more color and contrast, making buttons larger.

Phase 1: Patient Participant Demographics

White (n=14, 70%) African-American (n=2, 10%)

Native Hawaiian/Pacific Islander (n=2, 10%) Asian (n=2, 10%)

Male (n=14, 70%) Female (n=6, 30%)

Age Range: 40 – 82

Findings – Patients

- Comfort with using apps or technology (5 point scale)
  - *Means*, All: 3.3 (Madigan: 2.8 , Nellis: 3.8)
- Task completion times
  - *Range*: 3.29 sec - 249.45 sec
- Single Ease Use Question (7 point scale)
  - *Range of Means:* 2.8 – 6.6
- System Usability Scale (SUS)
  - SUS overall*, Mean (SD)*: 83.8 (14.9) = A+
  - Usability Sub Factor*, Mean (SD)*: 86 (13.4) = A+
  - Learnability Sub Factor*, Mean (SD)*: 75 (27.5) = A-

Findings – Clinicians

- Overall, Clinicians were pleased with the mCare system and optimistic about both the backend portal ("doctor's view") and application utility for patients.

- Clinicians also had additional suggestions specifically related to alerts, e.g., parameters for alerts sent to patients, color coding alerts for ease of clinician review.

- Patient and Clinician suggestions were reviewed and incorporated as adaptations by the technology team as allowed by system constraints.

Phase 1: Clinician Demographics

19 Physicians (2 DO, 17 MD) / 1-19 years experience

14 Clinical staff (FNP, nurses, disease managers, pharmacists) / 4-39 years experience

Recommendations to TATRC

- 29 specific recommendations were made to TATRC
  - Only 4 could NOT be completed
    - additional colors (limited colors available)
    - remove signal and refresh buttons (part of the base system)
    - alert icons (limited icon choices available)
    - automatic syncing of devices (system cannot do this)
  - >86% of recommended changes WERE made

Usability testing was CRITICAL in understanding the needs of end users and provided a more meaningful interface for Phase 2 participants

TATRC Modifications

Patients

- increase size of icons
- improved glucose graph responsiveness
- increase font size
- allow past dates for manual entry

Clinicians

- change default blood glucose entry to unclassified
- define "after meal" glucose as $\geq$120 minutes post meal
- simplify safety alerts (with Clinical Advisory Team)
    - add icons beside safety alerts
    - add patient target ranges on graphs
    - Clinicians can modify safety alerts
