# Supplementary material for: Enhancing Patient Activation and Self-Management Activities in Patients With Type 2 Diabetes Using the US Department of Defense Mobile Health Care Environment: Feasibility Study
Source: J Med Internet Res. 2020 May 26;22(5):e17968. doi: 10.2196/17968 (PMC7284404; doi:10.2196/17968)
Supplement: Multimedia Appendix 6 [file jmir_v22i5e17968_app6.pdf]

Enhancing mHealth Technology in the PCMH Environment to Activity Chronic Care Patients
Contract #W81XH-15C-0070

Clinical Data Standards, Alerts and Safety Algorithms Report

March 15, 2016

Ronald Gimbel, PhD, Principal Investigator

Appendix A

Measure: Blood Glucose Clinical Thresholds and Alerts

Glucose and Hypoglycemia- The main sugar that the body makes from the food in the diet. Glucose is carried through the bloodstream to provide energy to all cells in the body. Cells cannot use glucose without the help of insulin. Hypoglycemia is characterized by a reduction in plasma glucose concentration to a level that may induce symptoms or signs such as altered mental status and/or sympathetic nervous system stimulation. [1]

When blood glucose measure >300 mg/dl [1-3]

Importance of threshold: Evidence suggests that blood sugar levels >300 may result in severe health consequences and may require immediate medical attention

Action(s): Patient tailored message sent: If your blood sugar is greater than 300 mg/dl, drink water, be active, and check your blood sugar before meals and at bedtime until it is less than 200. If it does not go down contact your doctor.

PCMH Team: Alert sent to PCMH representative (site specific)

When blood glucose measure >250 mg/dl [2]

Importance of threshold: Evidence suggests that blood sugar levels >250 may result in severe health consequences and may require immediate medical attention

Action(s): Patient tailored message sent: Contact your doctor if your blood sugar is over 250 mg/dl for 24 hours.

PCMH Team: Alert sent to PCMH representative (site specific)

When blood glucose measure <100 mg/dl [2]

Importance of threshold: Evidence suggests that blood sugar levels <100 may result in severe health consequences and may require immediate medical attention

Action(s): Patient tailored message sent: If your bedtime blood sugar is less than 100 mg/dl, have a snack such as a glass of milk or a half a sandwich.

PCMH Team: Alert sent to PCMH representative (site specific)

When blood glucose measure <70 mg/dl [2]

Importance of threshold: Evidence suggests that blood sugar levels <70 may result in severe health consequences and may require immediate medical attention

Action(s): Patient tailored message sent: If your blood sugar is less than 70 mg/dl, treat with 3 glucose tablets, ½ glass juice, or other snack containing sugar. Recheck blood sugar after 15 minutes. If still less than 70, eat another 3 glucose tablets and recheck after 15 minutes. Repeat until blood sugar is above 70 mg/dl. After your blood sugar is back above 70 mg/dl, eat a small snack if your next meal is more than ½ hour away.

PCMH Team: Alert sent to PCMH representative (site specific)

Appendix B

Measure: High Blood Pressure Clinical Thresholds and Alerts

Hypertension - A condition present when blood flows through the blood vessels with a force greater than normal. Also called high blood pressure. Hypertension can strain the heart, damage blood vessels, and increase the risk of heart attack, stroke, kidney problems and death. [(4)]

For systolic blood pressure above 180:

Importance of threshold: Evidence suggests that systolic blood pressure above 180 may result in severe health consequences and may require immediate medical attention. [(4-7)]

Action(s): Patient tailored message sent: First Send: If you have chest pain, shortness of breath, back pain, numbness/weakness, change in vision or difficulty speaking, call 911 immediately. **Then send:** For systolic blood pressure above 180, wait 10 minutes at rest and recheck. If it is still above 180 systolic (the top number) and you do not have any other symptoms, contact your doctor.

PCMH Team: Alert sent to PCMH representative (site specific)

For diastolic above 110:

Importance of threshold: Evidence suggests that diastolic blood pressure above 110 may result in severe health consequences and may require immediate medical attention [(4-7)]

Action(s): Patient tailored message sent: For diastolic above 110 send this first: If you have chest pain, shortness of breath, back pain, numbness/weakness, change in vision or difficulty speaking, call 911 immediately. Then send this: For Diastolic blood pressure above 110 wait ten minutes at rest and check again. If it is still above 100 diastolic (the bottom number) and you do not have any other symptoms, contact your doctor.

PCMH Team: Alert sent to PCMH representative (site specific)

Appendix C

Measure: Weight Gain related to Coronary Heart Failure Clinical Threshold

Coronary Heart Failure and Weight Gain – Heart failure is a condition in which the heart is no longer able to pump oxygen-rich blood to the rest of the body efficiently. This causes symptoms to occur throughout the body. Weight gain, especially over a day or two, can be a sign that your body is holding on to extra fluid and your heart failure is getting worse. Talk to your doctor about what you should do if your weight goes up or you develop more symptoms. [(8)]

Weight gain of greater than 5 pounds in one week:

Importance of threshold: Evidence suggests that weight gain of at least five pounds in one week may be an indication of coronary heart failure and may require immediate medical attention [(9-10)]

Action(s): Patient tailored message sent: If you note a weight gain of greater than 5 pounds in one week, or you notice increased leg swelling or new or increased shortness of breath, call your doctor.

PCMH Team: Alert sent to PCMH representative (site specific)

Appendix D

Measure: Activity Monitor/Fit Bit Clinical Thresholds

Fit Bit – device that contains a 3D motion sensor that accurately tracks your calories burned-steps taken-distance traveled and sleep quality [11]

If no fit-bit activity in 24 hours:

Importance of threshold: Evidence suggests that stopping daily activity may result in health concerns and may require immediate medical attention

Action(s): Patient tailored message sent: If you need assistance with your blue tooth enabled fit bit to monitor your activity, please contact the research associate.
PCMH Team: Alert sent to PCMH representative (site specific)

Appendix E

Clinical Advisory Team Members

- **Lt. Col. Paul F. Crawford, III, MD, MOFMC - Chair**
- **Ron Gimbel, PhD, Clemson**
- **COL Eric A. Shry, MD, MAMC**
- **Liwei Chen, MD, MPH, Clemson**
- **Windsor Sherrill, PhD, Clemson**
- **Cheryl Dye, PhD, Clemson**
- **Terry Newton, MD, OTSG**
- **COL David P. Vetter, MD, MAMC**

Appendix F

Development Advisory Team Members

- **Jeanette Little, TATRC - Chair**
- **Holly Pavliscsak, TATRC**
- **Terry Newton, MD, OTSG**
- **COL David P. Vetter, MAMC**
- **COL Eric Shry, MAMC**
- **LtCol Paul Crawford, MOFMC**
- **Joel Williams, PhD – Clemson**
- **Lu Shi, PhD – Clemson**
- **Ron Gimbel, PhD Clemson**

Appendix G - References

1. Piette, J. D. (2008). QUERI Researchers Evaluate Automated Telephone Assessment and Patient Education as a Strategy for Improving VA Diabetes Care. PsycEXTRA Dataset, 54-68. Retrieved from http://www.queri.research.va.gov/tools/diabetes/shared-med-appt.pdf

2. Blood glucose. (2012, April). Retrieved March 07, 2016, from http://www.medicinenet.com/script/main/art.asp?articlekey=32858

3. Hamdy, O., MD, & Khardori, R., MD. (2015, September). Hypoglycemia. Retrieved March 07, 2016, from http://emedicine.medscape.com/article/122122-overview

4. Eisenberg, J. (2012, February). High Blood Pressure. Retrieved March 07, 2016, from http://www.ncbi.nlm.nih.gov/pubmedhealth/PMHT0024199/

5. Varon, J., Elliott, W. Evaluation and treatment of hypertensive emergencies in adults. In: UpToDate, Post, TW (Ed), UpToDate, Waltham, MA, (Accessed on March 1, 2016)

6. Kessler, C., Joudeh, Y. (2010). Evaluation and Treatment of Severe Asymptomatic Hypertension. *American Family Physician, 8*(4), 470-476

7. American Heart Association (2015, October 22). Hypertensive Crisis. Retrieved from www.heart.org

8. Colucci, W. S., MD. (2015, December). Patient information: Heart failure (Beyond the Basics). Retrieved from http://www.uptodate.com/contents/heart-failure-beyond-the-basics

9. What Is Heart Failure? (2015, November). Retrieved March 07, 2016, from http://www.nhlbi.nih.gov/health/health-topics/topics/hf

10. Chen, M. A., MD. (2015, April 20). Heart failure - overview: MedlinePlus Medical Encyclopedia. Retrieved March 07, 2016, from https://www.nlm.nih.gov/medlineplus/ency/article/000158.htm

11. Wachsman, D. (n.d.). Fitbit. Retrieved March 07, 2016, from http://www.collinsdictionary.com/submission/10039/Fitbit
